# Hampering the early aggregation of PrP-E200K protein by charge-based inhibitors: a computational study

**DOI:** 10.1007/s10822-021-00393-7

**Published:** 2021-06-10

**Authors:** Mariangela Agamennone, Loriano Storchi, Alessandro Marrone, Roberto Paciotti

**Affiliations:** 1 Department of Pharmacy, University “G d’Annunzio” of Chieti-Pescara, Chieti, Italy; 2 Molecular Discovery Limited, Middlesex, London, UK

**Keywords:** Charge-based inhibitors, PrP-E200K, Molecular docking, Virtual screening, Anti-prion drugs

## Abstract

A multilayered computational workflow was designed to identify a druggable binding site on the surface of the E200K pathogenic mutant of the human prion protein, and to investigate the effect of the binding of small molecules in the inhibition of the early aggregation of this protein. At this purpose, we developed an efficient computational tool to scan the molecular interaction properties of a whole MD trajectory, thus leading to the characterization of plausible binding regions on the surface of PrP-E200K. These structural data were then employed to drive structure-based virtual screening and fragment-based approaches to the seeking of small molecular binders of the PrP-E200K. Six promising compounds were identified, and their binding stabilities were assessed by MD simulations. Therefore, analyses of the molecular electrostatic potential similarity between the bound complexes and unbound protein evidenced their potential activity as charged-based inhibitors of the PrP-E200K early aggregation.

**Supplementary Information:**

The online version contains supplementary material available at 10.1007/s10822-021-00393-7.

## Introduction

Since its proposal by Prusiner [1, 2], the prion’s concept have entered in the body of knowledge of several fields of Chemistry and Biology, and several neurodegenerative diseases are currently categorized to be caused by prions or prion-like proteins [3]. Nowadays, the prion’s concept is fundamental to a deep understanding of the pathogenesis of amyloid diseases, not only due to the often-similar phenotypical profiles, but also caused by common structural events affecting the involved protein such as acquired self-aggregation propensity, unfolding to a beta-rich conformation, and formation of neurotoxic aggregates [4–7]. The human prion protein (PrP), the causative agent of severe amyloid neurodegenerative diseases [8], is physiologically expressed in several tissues and characterized by basically an unstructured N-terminal domain (23–124), and a structured or globular C-terminal domain (125–231) [9]. Several pathogenic mutants of PrP have been recognized to be causative of familial forms of prion diseases, all characterized by a sudden inset leading to rapid decline of neurological functions and, eventually, to death [10–13]. Among them, the E200K mutation of PrP is recognized to be the cause of the most common familial form of the Creutzfeldt-Jakob disease (CJD) [10]. Both heterozygous and homozygous mutants have been investigated to elucidate the pathogenic role played by E200K, especially to discern among loss-of-function or attainment of new protein functions [14].

The tridimensional structure of PrP-E200K in water solution characterized by means of NMR approaches has been shown to be substantially similar to the wild type one, the only worth unveiled difference consisting in the distribution of surface charges [15]. Therefore, several studies have evidenced that an E200K-like pattern of surface charges may be induced in the wild type through the interaction with Ca^2+^ ions [16–18].

In the attempt of deepening the understanding of the structural features induced by E200K mutation, we have recently developed a computational workflow for the analysis of the molecular properties of protein systems based on the human PrP as a case study [17–19].

The analysis of the PrP-E200K structure compared to the wild type (wt) unveiled an intrinsic propensity of the mutant to aggregation, because its surface charge distribution (not the wt one) is seemingly capable to drive the long-range interaction of protein units [17–19].

Several lines of evidences have confirmed the importance of pre-fibril aggregates, such as oligomers, in the pathogenesis of prion-based diseases, although the way these aggregates express their harmful potential is still widely debated [20, 21].

**Fig. 1:**
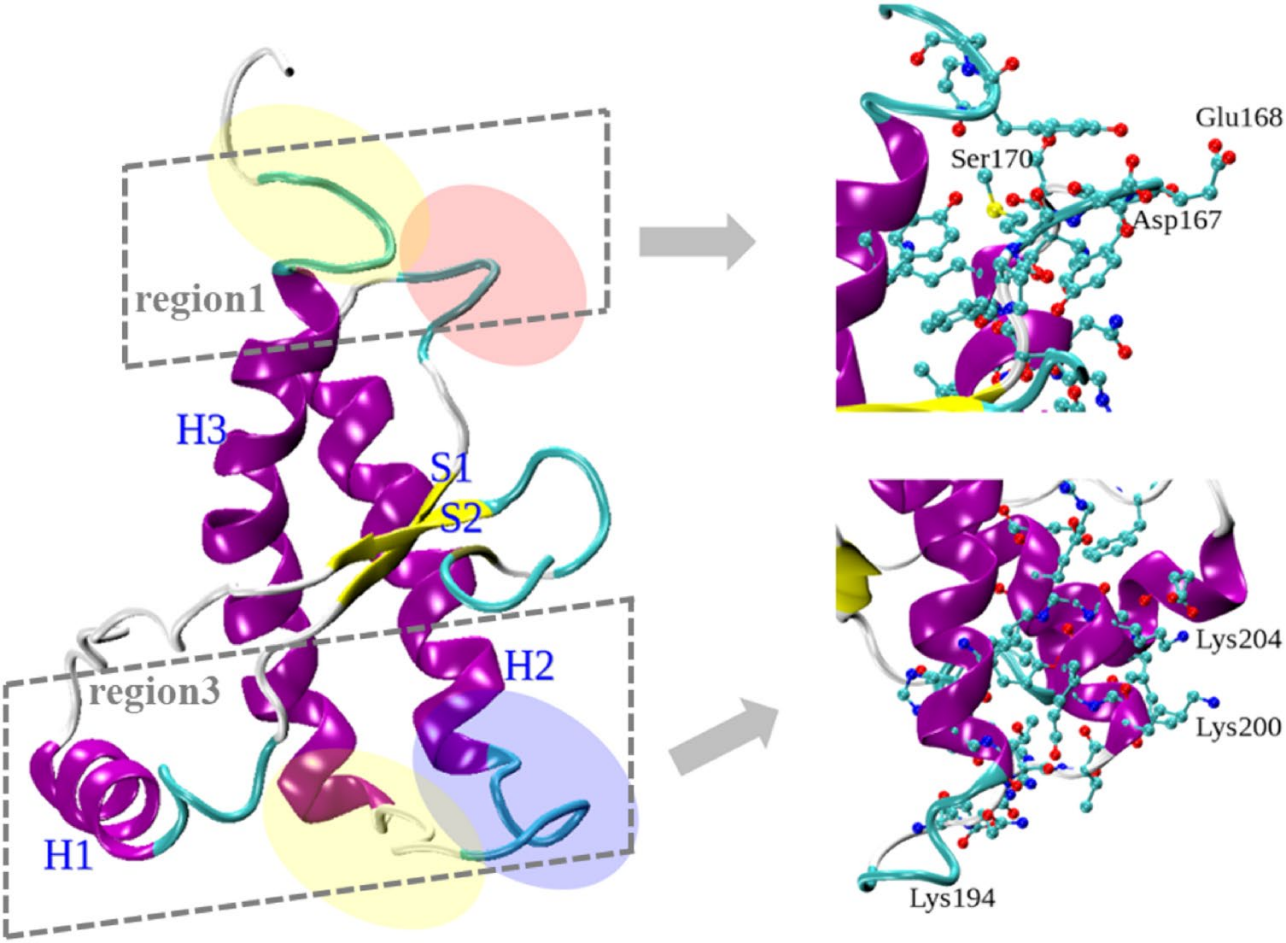
Representation of the 120–231 segment of PrP-200K protein displaying the determinant features involved in the early aggregation. Left: cartoon representation of the protein and the major positively (blue) or negatively (red) charged regions, together with hydrophobic spots (yellow). Region 1 and Region 3 are also delimited (dashed lines). Right: cartoon representation of Region 1 (top) and Region 3 (down) with ball-and-sticks models (hydrogen not shown) of the most significant residues in the protein–protein interactions

In our previous studies, as shown in Fig. 1, two portions of PrP-E200K were emerged to be majorly involved in its early aggregation:

- Region 1: mainly comprising the protein portions located nearby the C-terminus and characterized by a negative charge and exposure of hydrophobic residues; this region is not appreciably affected by the E200K.
- Region 3: mainly comprising the C-terminal and N-terminal portion of helices H2 and H3, respectively, and including the mutated residue; this region is profoundly affected by the E200K mutation that essentially induces a positive charge, while the same region was found to be weakly negatively charged in the wt.

The electrostatic complementarity between Region 1 and Region 3 have been assumed to lead to an effective head-to-tail interaction between PrP-E200K units and to initiation of the aggregation process, where the protein monomers are still in the cellular folded conformation, PrP^C^-E200K—the expression “early aggregation” will be used here to denote aggregates of PrP^C^-E200K units to be distinguished from the amyloid aggregates of disease-associated scrapie proteins, PrP^Sc^-E200K. Based on these structural insights, we had afterward simulated the structure of the PrP-E200K dimer, and performed quantum chemical calculations by the use of the fragment molecular orbital (FMO) approach to corroborate the involvement of Region 1 and 3 in the aggregation of PrP-E200K, and spotlight the role played by single protein residues [19]. The molecular dynamics (MD) simulations reinforced the postulated Region 1–Region 3 interaction in the dimer structure, and, also, unveiled that this protein–protein assembly induces some slight but detectable conformational adaptation, mostly in the unit interacting via Region 3 [19]. FMO calculations then provided for estimates of both the inter- and intra-domain residue-residue interaction energies, and showed the H2–H3 loop in unit A and the S2–H2 loop in unit B hosting the most stable inter-unit interactions, and, concomitantly, assuming a beta-sheet domain structure.

**Fig. 2:**
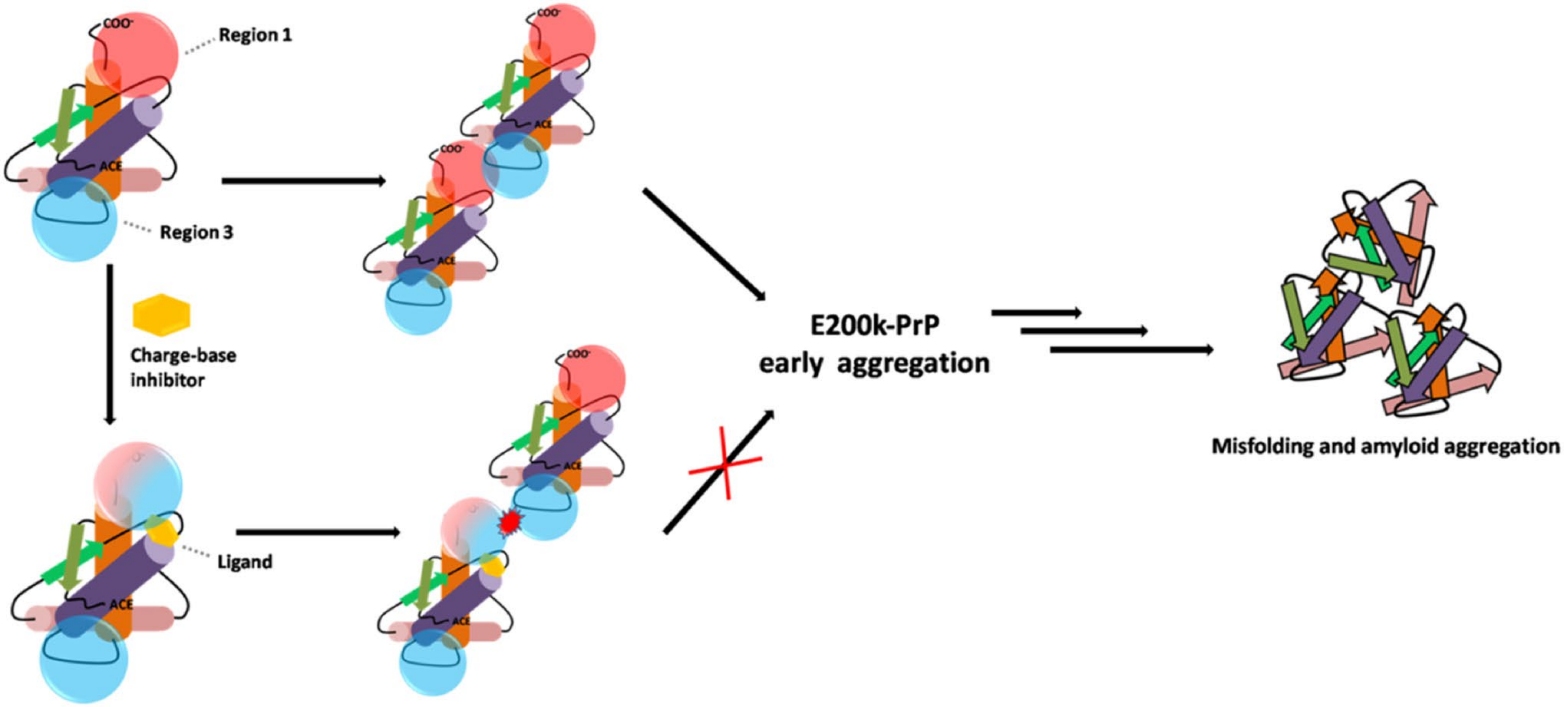
Mechanism of action of charge-based inhibitor as anti-prion drug. Such ligand should be able to disrupt the charge complementarity between two PrP-E200K units, hampering the early aggregation process. The negatively charged Region 1 is represented by red sphere while the positively charged Region 3 is represented with cyan sphere

The mentioned outcomes of QM and MD analyses on the model of PrP-E200K dimer provided a basis of detailed information on the approach of two protein units during the early stages of aggregation. On the other hand, the possible intervention on tempering or hampering the aggregation propensity of PrP-E200K, through the binding of small molecules able to disrupt the charge–charge complementary lasts as an appealing but still unexplored hypothesis. Such molecules are also known as charge-based inhibitors and their mechanism of action is schematized in Fig. 2 [22].

Many studies have been carried out in order to find small molecules with an anti-prion activity [23]. Promising molecules were characterized by different chemical scaffolds but all share the presence of one or more heterocyclic ring such as Congo red [24, 25], suramin [26, 27], curcumin and its derivatives [28–31], quinacrine, quinoline, acridines, and phenothiazines [32–35] to cite some of them. An exhaustive description of anti-prion drugs has been recently proposed by Barreca et al. [36, 37].

Computational approaches have been also used to support the discover of new promising anti-prion molecules. Recently, Kuwata and co-workers have employed a specific computational procedure, named NAGARA, for the design of a new molecule acting as pharmacological chaperone, being able to bind PrP^C^, and blocking the prion conversion and replication not only in cultured cells but also in infected animals [38–40]. Other computational approaches have been applied in order to find new anti-prion ligands with promising results [41], hence, suggesting that computational chemistry can boost the research of new drugs also in this field.

**Fig. 3:**
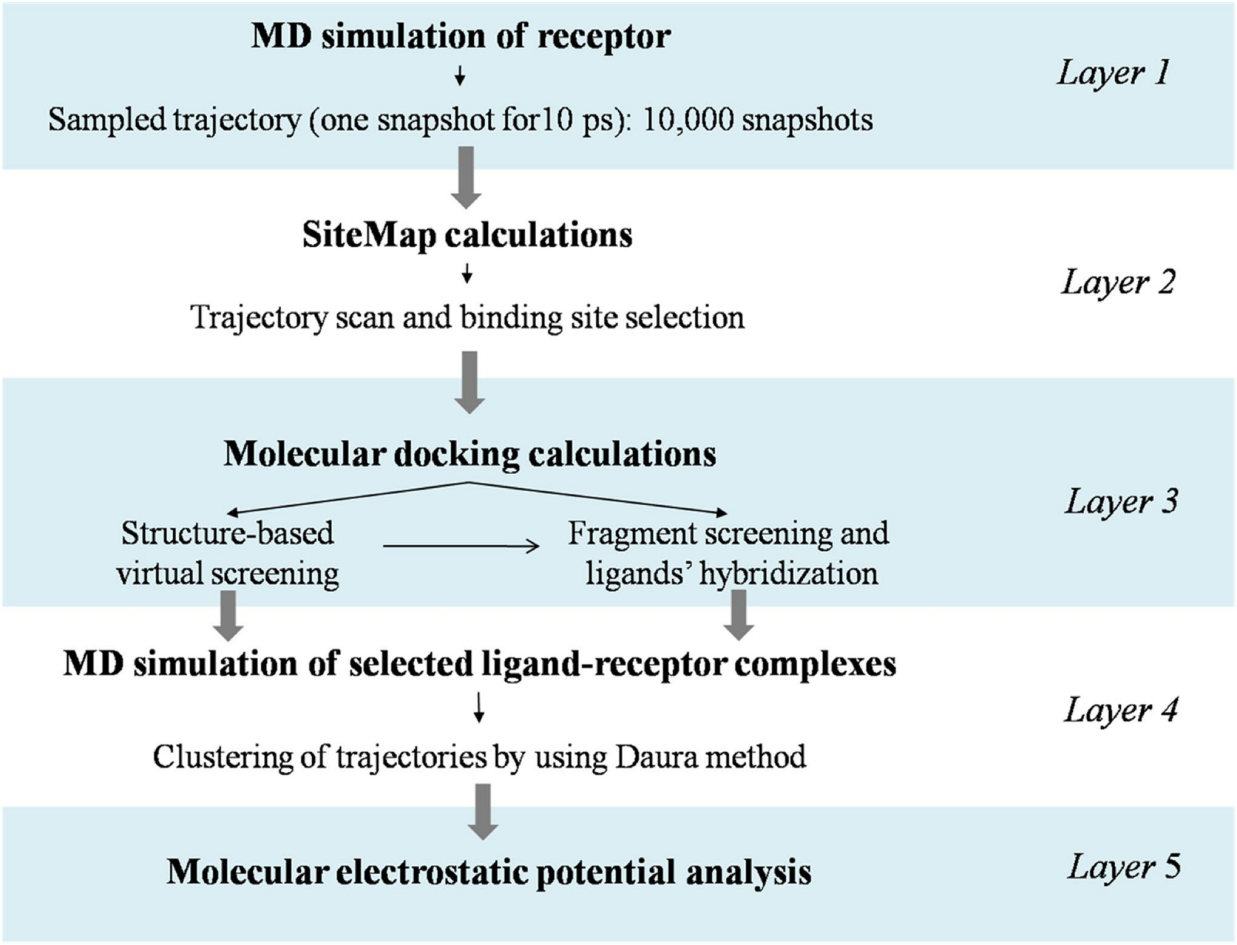
Schematic representation of multi-layered method applied in this work. *Layer 1* MD simulation of receptor to explore the conformational space of the protein and to collect a great number of protein conformations. *Layer 2* SiteMap calculations on the protein conformations collected on layer 1 to identify promising binding pockets. SiteMap results are refined by considering the geometrical parameters of the binding pocket, as sphericity and planarity, and the final set of binding sites were defined. *Layer 3* structure-based virtual screening, fragment screening and ligands’ hybridization to identify the best ligands. *Layer 4* MD simulation of ligand-receptor complexes identified in the previous layer and the corresponding trajectories were clusterized by using Daura’s method. *Layer 5* molecular electrostatic potential (MEP) calculations to assess the impact of the detected binders on the electrostatic potential of PrP-E200K

In this study, we applied our multilayered computational workflow, summarized in Fig. 3, to investigate the effect of the binding of small molecules at the PrP-E200K in seeking for potential inhibitors or modulators of this prion protein aggregation. The inhibition of the early aggregation of PrP-E200K may represent a promising approach to prevent the formation of prion oligomers, and, consequently, all the associated downhill processes.

Although the targeting of the PrP-E200K resulted to be quite challenging due to the lack of unambiguously documented binding pockets, our study provided a computational procedure (Fig. 3) to impel the search of targetable conformations of this protein through an extensive screening of the MD trajectory, and eventuated with the identification of a suitable binding site. Following structure-based virtual screening of a diverse library and a fragment-based approach afforded small molecules hitting the identified site. MD simulation of the obtained putative complexes confirmed the stability of the binding and better defined the interaction patterns. The molecular electrostatic potential (MEP) of bound complexes with top ranked ligands were also analyzed and compared allowing us to quali-quantify the selected ligands in terms of their potential activity as charged-base inhibitors of the PrP-E200K early aggregation.

## Methods

### MD simulations

The molecular dynamics procedure for the simulation of the 120–231 segment of PrP-E200K (Fig. 1), performed with the Gromacs package [42], had been widely described in previous studies [18]. Briefly, the protein molecule was placed in a cubic box (7.3805 nm), solvated with TIP3 [43] water molecules (12,577) at the typical room-temperature density of liquid water (1.0 g·cm^3^), and added by one Cl^–^ counterion to gain electrical neutrality. The protein system was simulated in the OPLS-2005 force field [44, 45] within the following scheme: (i) local energy minimization; (ii) slow heating up: preparation runs (200 ps) with the protein temperature increasing by 50 K from 150 to 300 K; (iii) production runs of 300 ns at 300 K. All simulations were performed in an isothermal/isobaric ensemble, using the velocity rescaling scheme (temperature) and the isotropic Berendsen coupling scheme (pressure) [46]. The LINCS constraining algorithm was adopted [47], and the long-range electrostatics were computed by the Particle Mesh Ewald method [48]. The last 100 ns segment of production run trajectory was sampled by extracting one snapshot every 10 ps, thus, forming a set of 10,000 protein conformations used as input structures for subsequent binding pockets searching.

A strictly analogous computational procedure was employed for the simulation of the complexes between PrP-E200K protein and ligands identified by the fragment-based approach (vide infra). The OPLS topologies of the investigated ligands was generated using *Acpype* [49, 50], a tool based in Python to use Antechamber [51, 52]. The only relevant differences are represented by a first thermalization run of 10 ns in NVT conditions and then a production run of 200 ns in NPT conditions.

For each ligand–protein complexes, the production run trajectory was sampled by extracting one snapshot per 100 ps, thus, forming a set of 2000 ligand–protein complexes on which all further analyses were performed by using suitable Gromacs utilities with the support of either VMD or Maestro graphical interfaces [53, 54]. In details, 500 ligand–protein conformations, corresponding to the last 50 ns segment of sampled production run trajectory, were used as input structures of cluster analysis performed by the employment of the *g_cluster* utility through the Daura’s method [55], labeled *gromos* and implemented in Gromacs package. A clustering cut-off of 0.19 nm was employed in the comparison of sampled MD snapshot based on the position of the ionizable residues, as reported in previous studies [17–19],

As results, we obtained for each ligand–protein complex a set of middle elements of the clusters, where one refers to “middle element” as the structure with the smallest average RMSD from all other structures of the same cluster. Finally, the structures corresponding to middle elements, covering at least 95% of the whole clusters ensemble, were selected to generate the representative structures subset of each ligand–protein system. Additional information and more detailed description of clustering procedure can be found elsewhere [17, 55].

Then, the representative structures of ligand–protein complexes (middle elements) were optimized with the MacroModel software [56] in the OPLS force field [44, 45] and the GB/SA polarizable continuum method [57, 58]. After alignment on the same principal axis, the optimized structures were suitable to MEP analyses.

### Screening of binding pockets

The 3D structures of the fragment 90–231 of PrP-E200K with PDB ID 1FO7 [59] was retrieved from the Brookhaven Protein Data Bank (PDB). 3D coordinates have been obtained by solution NMR and 30 conformers are reported in the structure file. All conformations were aligned to each other exploiting the Superposition Tool available in Maestro [60]. After the alignment, all structures were submitted to the Protein Preparation routine to fix the bond order and assign the correct protonation state to each residue, and, eventually, relax the protein structure by applying the default parameters. SiteMap calculations were carried out on each protein conformer to search for the five top ranked binding sites. A fine grid was calculated by using a more restrictive definition of hydrophobicity and sites cropped at 4 Å from the nearest site point.

### Trajectory scan and binding site selection

The present section describes the approach we implemented to extract the site assessed in the subsequent virtual screening campaign against the PrP-E200K receptor. At this purpose, the last segment of MD trajectory (100 ns) was skipped each 10 ps to obtain an ensemble of 10,000 snapshots to allow the exploration of short-lived binding sites.

The localization of the binding sites on each one of 10,000 protein conformations and the prediction of their druggability were performed with SiteMap, by applying the same parameters described in the previous paragraph [61]. Such a process is highly parallelizable; indeed each binding site search per conformational state is independent. Thus, we developed a simple Python script [62] to automatize both the search as well as the subsequent sites extraction.

The standard druggability indices computed by SiteMap, i.e., SiteScore, exposure, enclosure, and balance, plus a set of geometrical parameters, such as the planarity and sphericity, were taken into account to score the analyzed binding sites.

The geometrical information, i.e., planarity and sphericity, can be easily computed by performing the principal component analysis (PCA) on the covariance matrix of the grid points constituting a site. Three eigenvalues (λ_1_, λ_2_, λ_3_) and the corresponding eigenvectors were gained, thus describing the variation of the points constituting the site around the center (i.e., the site centroid) along with their principal directions (three orthogonal vectors), and their magnitudes (eigenvalues). Hence, sphericity and planarity indexes can be easily defined as follow:

1 $${\text{S}}_{\lambda } = {{\lambda _{{\text{3}}} } \mathord{\left/ {\vphantom {{\lambda _{{\text{3}}} } {\lambda _{{\text{1}}} }}} \right. \kern-\nulldelimiterspace} {\lambda _{{\text{1}}} }}$$

2 $${\text{P}}_{\lambda } = {{\left( {\lambda _{{\text{2}}} - \lambda _{{\text{3}}} } \right)} \mathord{\left/ {\vphantom {{\left( {\lambda _{{\text{2}}} - \lambda _{{\text{3}}} } \right)} {\lambda _{{\text{1}}} }}} \right. \kern-\nulldelimiterspace} {\lambda _{{\text{1}}} }}$$

where S_λ_ is the sphericity and P_λ_ the planarity index respectively [63].

A two-stage approach was employed to filter out and to score the binding sites localized by SiteMap: (i) we firstly retained all binding sites with SiteScore > 0.8, exposure < 0.49, enclosure > 0.78 and balance ≥ 1.6 (i.e. all those parameters are directly computed by the SiteMap software  [64]); (ii) we secondly retained all the sites with a sphericity and planarity greater than their respective average values, considering that the volume of a binding site should be equal or higher than that one of a ligand, and with a shape enabling the ligand to fit in the binding site [65].

Such a procedure ended up with a reduced set of 36 binding sites, ranked according to SiteScore values, which could be easily visualized and inspected. The superposition of the identified binding sites on the PrP-E200K ensemble allowed a straightforward delimitation of binding regions (BRs) that can be defined as space portions hosting a binding site in more than one conformer (MD snapshot): the number of binding sites located in a BR can be assumed as a measure of its residence time and, thus, it quantifies its relevance. Each binding site located in a certain BR of a MD snapshot was labelled with the number of corresponding trajectory frame (e.g., the binding site calculated on the frame 4204 of trajectory and located in BR1 is named BR1_4204).

### Molecular electrostatic potential analysis

As described above, the clustering of the last 50 ns of production trajectories provided for a set of representative conformations for each ligand-E200K complexes. Then, we performed a comparison of MEP similarity between free E200K and each representative conformation of bonded E200K, using a Carbò index evaluation, as described elsewhere [17]. The final profiles were plotted as the weighted average of Carbò index values, reporting the standard deviation values that yield an indication of the MEP similarity “stability” in a specific protein region.

In details, the MEP is calculated immersing the considered target structure in a three-dimensional grid, thus a positively charged probe (q =  + e) is moved in each point of the grid, and its Coulomb interaction energy with all protein atoms is computed [17–19].

Furthermore, the computed MEP can be used to compare two proteins along a specific axis using the Carbò index (CI), as described in our previous works [17–19]. Briefly, to assess the MEP similarity between two proteins along the X axis, the CI is computed as follows:

3 $$CI\left( {i_{x} } \right) = \mathop \sum \limits_{{j_{y} = 1,~~k_{z} = 1}}^{{N_{y} ,~~N_{z} }} ~~~\frac{{MEP_{1} \left( {i_{x} ~,~j_{y} ~,~k_{z} } \right)~ \cdot ~MEP_{2} \left( {i_{x} ~,~j_{y} ~,~k_{z} } \right)}}{{\sqrt {MEP_{1} \left( {i_{x} ~,~j_{y} ~,~k_{z} } \right)^{2} ~ \cdot ~MEP_{2} \left( {i_{x} ~,~j_{y} ~,~k_{z} } \right)^{2} } }}$$

where MEP_1_(i_x_, j_y_, k_z_) and MEP_2_(i_x,_ j_y_, k_z_) are the computed molecular interaction potentials for the two proteins at each grid point, defined by cartesian coordinates i_x_, j_y_ and k_z_. By definition, − 1.0 ≤ CI ≤  + 1.0 where − 1.0 and + 1.0 correspond to the minimum and maximum similarity, respectively. Finally, it is important to note that, since we collected sets of structures as result of a clusterization of a MD trajectory, the MEP similarity computation was defined to allow the pairwise comparison of proteins sets rather than single structures. MEP similarity profiles were calculated for all possible pairs of conformations within two sets and the corresponding weighted average was computed to obtain an overall similarity profile. Notably, the weight factors were obtained through the clustering of MD trajectory of the systems.

## Identification of potential binders

### Structure-based virtual screening

For the structure-based virtual screening campaign, the Glide Grid generation was applied to the identified site of PrP-E200K, i.e., BR1_4204, using the Receptor Grid Generation utility of Glide [60]. The center of the calculated grid was defined as the centroid of following residues: Tyr163, Arg164, Pro165, Met166, Asp167, Glu168, Tyr169, Ser170, Asn171, Asn174, Phe175 and Asp178. The default settings were applied for remaining parameters.

The diverse library used for the virtual screening campaign was generated starting from a set of compounds downloaded from the Zinc15 repository (https://zinc15.docking.org/) [66] among the “In stock” and “anodyne” subgroups and with 250 < MW < 500 and 0 < logP < 5. A set of 9,943,152 molecules in SMILES format were obtained and subsequently undergone to the diversity selection process. To this aim, Knime [67] was exploited to build a proper workflow (Fig. S1 in SI). The FingerPrintGen node of Canvas was used for hashed radial fingerprints generation applying the following parameters: atoms were distinguished by functional type, a 32-bit precision was applied and bits were filtered by ON/OFF frequency. The following node carried out the Canvas Dissimilarity selection by using the Soergel metric to calculate distance, the sphere selection method and by imposing a distance threshold of 0.5. A final set of 400,000 diverse compounds was obtained and then processed with LigPrep to generate 3D structures, tautomers, stereoisomers, and to ionize compounds using Epik at target pH 7.0 ± 0.4. A final set of almost 1,000,000 structures was then submitted to virtual screening.

The structure-based virtual screening was carried always exploiting Knime and setting up a workflow mimicking the Virtual Screening Workflow of Maestro (Fig. S2). Three Glide grid nodes corresponding to HTVS (high-throughput virtual screening), SP (standard precision) and XP (extra precision) ligand docking were setup with a flexible treatment of ligands, performing post-docking minimization and saving 5 poses in the HTVS step and just one geometry in both SP and XP calculations. The 10% of top score compounds passed from HTVS to SP and from SP to XP, while the 20% of the best scoring virtual hits were saved and analyzed for final selection.

### Fragment screening and ligands’ hybridization

The Schrödinger fragment-library was docked in the prion binding site previously selected. The library comprises 441 unique fragments and 667 structures considering also tautomers and protomers. Glide SP docking was carried out and best 50% of SP docked structures passed to XP. Finally, best scored fragments were selected on the basis of docking score values, glide ligand efficiency, and, in particular, focusing on complementarity to the SiteMap molecular interaction fields (MIFs).

The 10 best fragments selected by docking were manually hybridized with the side chains of three virtual hits retrieved from the diverse screening. A library of 33 compounds was generated, submitted to LigPrep as previously described, and docked in the PrP-E200K binding site applying both SP and XP protocols to all ligands.

### Pharmacophore hypothesis generation

The Develop Pharmacophore hypothesis module of Phase [60] was used starting from the docked poses of compounds **1**–**6** in the PrP-E200K. Each binding pose was analyzed and features corresponding to identify interactions were manually selected obtaining a hypothesis for each complex. Eventually, all hypotheses were merged in a unique pharmacophore accounting for contacts conserved in at least 3 out of 6 ligands. Resulting hypothesis was used to align vidarabine structure, built in Maestro and submitted to generation of a maximum of 50 conformers using the ConfGen module.

## Results

### Binding site identification

The identification of suitable binding sites to host small molecules on the PrP-E200K structure was preliminarily carried out on the experimentally determined 30 NMR structures of this protein (PDB ID 1FO7) [59] using SiteMap as described in the “Method” section.

Such an analysis allowed the identification of several possible binding regions on the protein surface; however none of them presented a good profile of binding features with respect to the parameters suggested from the SiteMap manual (SiteScore > 0.80, exposure < 0.49, enclosure > 0.78, balance ≥ 1.6). Although the analysis on the experimental structures of PrP-E200K corroborated the presence of a potential binding pocket located between H2 and S1, this site was affected by high solvent exposure, and reduced accessibility of hydrophobic residues (Table S1). However, due to the structural flexibility of proteins, this binding pocket may assume several conformations where a potentially more druggable profile could be reached.

In this line, the molecular dynamics simulation of the 120–231 segment of PrP-E200K previously performed [18], was analyzed by the sampling of trajectory snapshots through the application of a filter developed to selectively identify and quantify suitable druggable pockets. At this purpose, 10,000 frames of trajectory underwent to an automated SiteMap analysis that yielded more than 21,000 possible binding sites. The application of selection criteria based on both SiteMap parameters and geometrical descriptors (sphericity and planarity) allowed identifying a final set of only 36 sites (Table S2).

The score values computed by SiteMap for the 36 selected sites are in better agreement with the SiteMap manual suggested scores for druggable sites with respect to those computed for binding sites detected on NMR conformations. In particular, more promising values have been obtained for exposure and enclosure parameters, while SiteScore and phobic/philic balance values are favorable also for NMR derived sites.

Indeed, enclosure and exposure represent two descriptors that evaluate how much the site is open to the solvent, where high (> 0.78) and low (< 0.49) values of enclosure and exposure, respectively, are repute to be better for a tight-binding site. Considering that E200K protein do not present buried hydrophobic cavities but instead small and quite solvent-exposed pockets, we considered the enclosure and exposure parameters as critical for the detection of promising hidden binding pockets. Indeed, in this view, none of the binding sites detected on NMR structures presented satisfactory values for those parameters, as shown in Table S1. On the contrary, the binding sites detected by trajectory scanning are characterized by the lowest exposure (range of 0.345 – 0.518) and the highest enclosure (range of 0.683 – 0.781) scores, very close to the reference values.

**Fig. 4:**
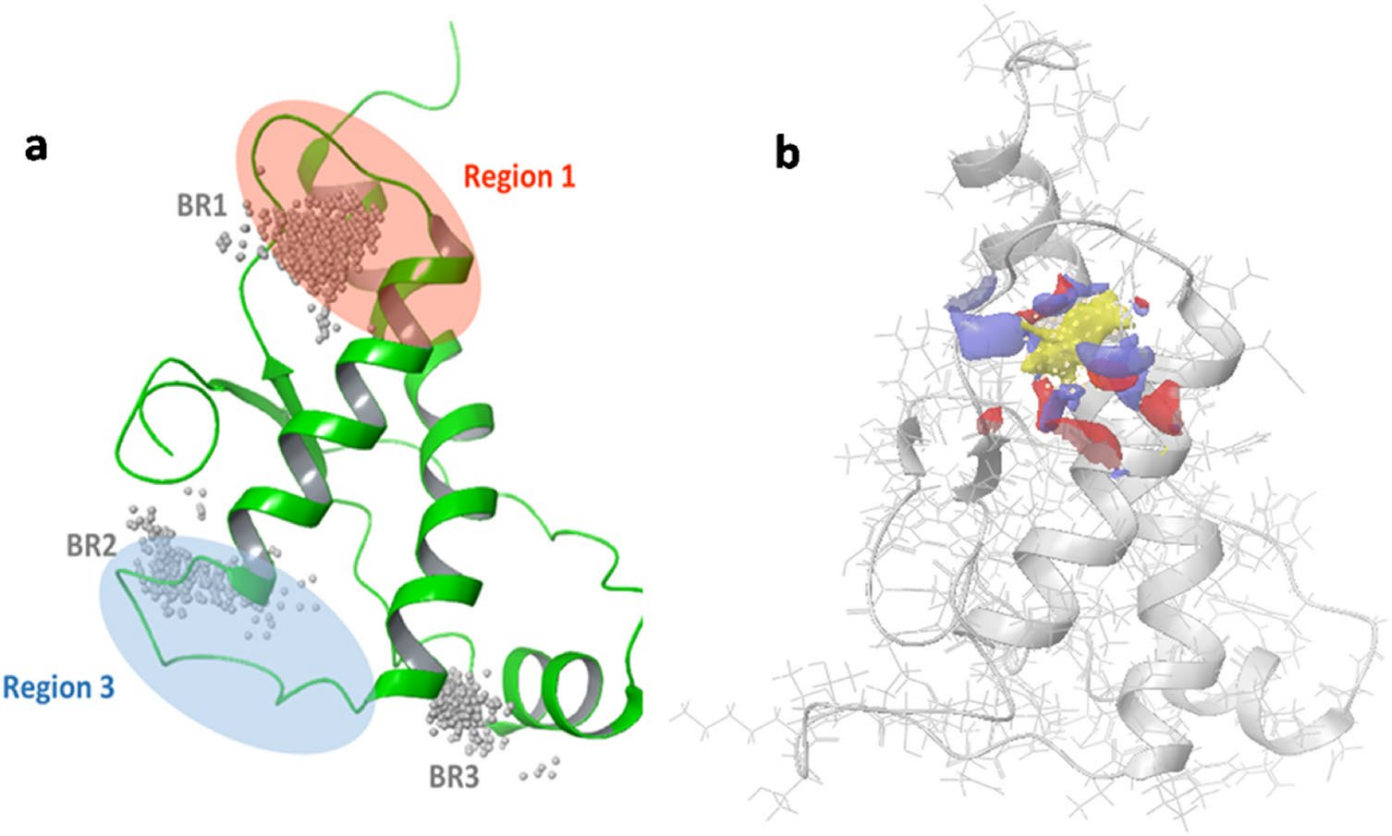
**a** Position of the three most persistent binding regions, named BR1, BR2 and BR3, identified on the PrP-E200K surface along with the location of Region 1 and 3, involved in our model concerning the early state aggregation of E200K protein; the site-point groups, calculated by SiteMap to locate the binding pocket, are represented as grey spheres. **b** Identified site, BR1_4204, with SiteMap molecular interaction fields (yellow surface = hydrophobic, blue surface = H-bond donor, red = H-bond acceptor), and site-points (white spheres)

As shown in Fig. 4a, three BRs, located in three different areas of the PrP-E200K surface, resulted to be characterized by the highest persistence (See “Methods”). Table 1 summarized the main features of the detected BRs. The most populated BR, named BR1, corresponds to the one already identified on the NMR structures and resides close to Region 1, between H2 and S1. Interestingly, BR1 includes residues Asp167 and Glu168 that majorly contribute to the negative charge of the Region 1. It is detectable in almost all analyzed frames of MD, demonstrating its high persistence in addition to its higher druggability.

**Table 1:** Potential binding regions (BR) identified on E200K surface and the main residues delimiting a druggable binding site

Binding region	Main residues defining druggable binding site	Note
BR1	Arg164, Pro165, Met166, Asp167, Glu168, Tyr169, Ser170, Asn171, Gln172, Asn174, Phe175, Pro177, Asp178	Most promising binding region; high druggability profile; high persistence; overlapping with Region 1
BR2	Gly124. Leu125, Gly126, Tyr128, Pro158, Tyr162, Gln186, His187, Val189, Thr190, Thr191, Thr192, Gly195, Glu196, Phe198	High druggability profile; diffuse and solvent exposed; average persistence; close to Region 3
BR3	Phe141, Ser143, Asp144, Tyr145, Glu146, Tyr149, Ty5150, Glu152, Asn153, Met154, His155, Arg156, Tyr157, Thr199, Thr201, Asp202, Lys204, Met205	Low druggubility profile; low persistence; close to Region 3

The second binding region, BR2, in the Region 3, is proximal to the C-terminal end of H2. The binding sites located in this region have been retrieved in the 67% of MD frames and some of them are reported among the best 36 selected sites from MD analysis. Despite good score values, the visual inspection of the best sites residing in this region showed a diffuse and solvent-exposed binding surface. The binding sites identified on BR3, also in Region 3 but located in the cleft formed by H1 and the N-terminal of H3, is represented in the 36% of the analyzed conformations. SiteMap evaluation of this site defines their poor druggability, in fact, no sites in this region are present in Table S2.

Among the three different BRs, the best scored sites were found in the BR1 and all of them present a hydrophobic core surrounded by H-bond acceptor and donor sites. As shown in Fig. S4a, the top scored sites, however, present a fragmented hydrophobic field with a portion residing outside the binding site. On the contrary, BR1_4204 shows a more defined and compact shape inside the binding pocket (Fig. 4b and Fig. S4b) and it is also characterized by the lowest exposure (0.345) and the highest enclosure (0.781) values suggesting a reduced solvent exposed binding surface. For this reason, this site was selected as the most promising binding site and used as target for a structure-based virtual screening campaign aimed to find some putative small-molecule binder of PrP-E200K.

### Diverse library virtual screening

The structure-based virtual screening campaign was carried out on a library retrieved from the ZINC15 database and comprising commercially available molecules with limited MW and logP values (250 < MW < 500 and 0 < logP < 5) to remain in the drug-likeness fields. The starting library of almost 10,000,000 compounds was submitted to a diversity-based selection to obtain a representative subset of the starting collection, in order to reduce the number of screened molecules and, consequently, the calculation time. A hierarchical docking-based screening was carried out on almost 1,000,000 structures affording a final set of 2,000 virtual hits. Top ranking compounds were carefully analyzed on the basis of their scores, in particular focusing on both docking score and glide ligand-efficiency. The latter parameter is calculated by dividing the glide docking score by the number of heavy atoms composing the ligand and provides a better indication of the effectiveness of the ligand interaction, where the docking score is usually proportional to the ligand molecular weight.

The final selection was carried out through visual inspection of the binding poses, by taking into account two main features: the overlap of docked compounds with molecular interaction fields calculated by SiteMap and the presence of functional groups, preferably positively charged, able to counteract the negatively charged residues surrounding the site. Three virtual hits, **1**–**3**, selected on the basis of these parameters are reported in Table 2.

**Table 2:** Ligand IDs, Zinc IDs, docking scores and molecular structure of selected virtual hits

Ligand ID^a^	Docking score	Glide ligand efficiency	Structure
1	− 10.087	− 0.420	
2	− 9.060	− 0.312	
3	− 6.815	− 0.235	

^a^Zinc IDs of the selected ligands: ZINC000257276435 (ligand **1**), ZINC000005487110 (ligand **2**) and ZINC000257209546 (ligand **3**)

All three compounds share a bicyclic-heteroaromatic scaffold bearing one or two small polar substituents, and a longer side chain presenting a saturated 5-membered heterocycle with hydroxyl substituents along with key alkylamine chains.

**Fig. 5:**
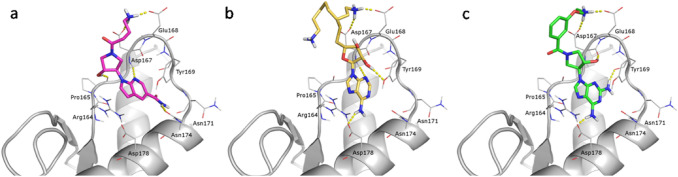
Binding poses of the selected virtual hits in the PrP-E200K (grey cartoon) BR1_4204 binding site. H-bonds are represented as yellow dashed lines. Ligands are shown as stick with differently colored C atoms: **a** ligand **1 **(violet), **b** ligand **2 **(yellow) and **c** ligand **3 **(green)

These common features provide for a similar pattern of interactions in the PrP^C^-E200K binding site, as shown by docked poses reported in Fig. 5: in particular, the protonated alkylamine group of all three ligands form salt bridges with both Asp167 and Glu168, demonstrating their potential as charge-based inhibitors. In details, **1** establishes two strong H-bonds with Asp167 and Glu168 by means of the positively charged alkylamine group. The 1H-pyrrolo[2,3-b]pyridine scaffold interacts with the aromatic ring of Tyr169 by $\pi - \pi$ stacking and the pyridine N forms a H-bond with Asp167 NH; the NH_2_ group interacts via H-bonds with Asn174 side chain and Asn171 backbone C=O, while the OH group of pyrrolidine is involved in H-bond with carbonyl group of Pro165.

Ligand **2** bears a tetrahydrofuran moiety with OH functions and two positively charged groups, but only one of the primary amino groups (on the shortest hydrocarbon chain) establishes H bonds with Glu168 and Asp167, resembling the ligand **1** docking pose. In the binding pose of **2** we found that the 9H-purin-6-amine scaffold is placed not totally inside the pocket and the alkylamine tails are totally solvent-exposed; the NH_2_ group in position 6 of the purine moiety forms a strong H-bond with Asp178 carboxylic group.

Finally, the 9-(pyrrolidin-3-yl)-9H-purin-2,4-diamine scaffold in ligand **3** allows the interactions with C=O group of Tyr169, with the NH of Asp167 and with the carboxylic moiety of Asp178, while the pyrrolidine OH establishes a H-bond with Asp167 C=O.

Most of the best scoring compounds retrieved from docking screening shares the same scaffold of compounds **1** and **3**.

Interestingly, all the three considered ligands of PrP-E200K contain cyclic aromatic moieties with N atoms, and anti-prion molecules with similar chemical features, although with different scaffolds, were previously detected using a yeast-based screening assay (e.g., phenanthridine and its 6-amino derivatives) [68].

### Fragment selection and ligand hybridization

The described binding poses of compounds **1**–**3** highlight the partial occupation and exploitation of possible interactions (mainly H-bonds) in the small binding pocket. Therefore, because of the limited diversity of the identified hit core, a scaffold hopping approach was applied to find a more tailored binder of our site, ensuring an optimized interaction with the binding site residues [69]. To this aim, the Schrodinger fragment library was docked in the BR1_4204. Best fragments were selected applying the same criteria described in the previous paragraph and considering, with particular attention, the glide ligand-efficiency as well as the matching with SiteMap calculated fields. A final set of 10 fragments was selected; their structures as well as their score values are reported in Table 3.

**Table 3:** Fragment ID, IUPAC name, docking scores and molecular structure of selected fragments

Fragment ID (IUPAC name)	Docking score	Glide ligand efficiency	Structure
**sf-285** (3-Metyl-7H-pyrrolo[2,3-d]pyrimidin-4-one)	− 7.930	− 0.721	
**sf-417** (1-Metyl-3,7-Dihydropyrido[3,4-d]pyrimidin-2,4,8-trione)	− 7.240	− 0.517	
**sf-282** (3-Hydro-7H-pyrrolo[2,3-d]pyrimidin-4-one)	− 7.035	− 0.704	
**sf-298a** (2H-pyrazolo[3,4-d]pyrimin-4-one)	− 6.970	− 0.697	
**sf-306** (1-Metyl-1H-pyrazolo[4,5-d]pyrimin-7-one)	− 6.513	− 0.592	
**sf-210** (Hexahydropyrimidin-2-one)	− 6.436	− 0.919	
**sf-335** (3,4-Dihydro-2H-isoquinolin-1-one)	− 6.344	− 0.577	
**sf-118** (1H-Pyrrolo[2,3-b]pyridine)	− 6.076	− 0.675	
**sf-28a** (Imidazolin-2,5-dione-4,4′-piperidinium)	− 6.013	− 0.501	
**sf-128** (1H-Pyrazolo[3,4-b]pyridine)	− 5.933	− 0.659	

*sf:* Schrodinger fragment

All the selected fragments are cyclic compounds containing one or more N atoms. The fragments with the best scores are aromatic compounds, structurally related to purine and guanine, as pyrrolo[1–3,d]pyrimidine (docking score: − 7.930) and pyrido[4–3,d]pyrimidine (docking score: − 7.240).

**Fig. 6:**
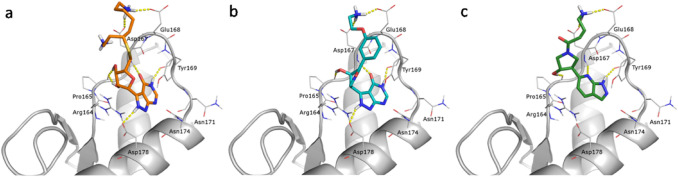
Binding poses of the selected virtual hybrid hits in the PrP-E200K (grey cartoon) BR1_4204 binding site. H-bonds are represented as yellow dashed lines. Ligands are shown as stick with differently colored C atoms: **a** ligand **4** (orange), **b** ligand **5** (cyan) and **c** ligand **6 (**green)

The original scaffold of the three virtual hits **1**–**3**, possessing the desirable charged side chains, were substituted with the selected fragments. Then, a new small library of 33 hybrid compounds was built attempting to maintain the correct orientation of both charged side chains and newly identified fragments. Finally, after docking calculations on BR1_4204 binding site, three new hybrid ligands, named **4**, **5** and **6**, were selected: their structures and docking scores are reported in Table 4 while their docking poses are shown in Fig. 6. More in details, ligands **4**–**6** are obviously strictly correlated with **1**–**3** but with significant differences. In particular, while the aromatic N-bicyclic scaffold of **1**–**3** are connected with 5-membered ring (tetrahydrofuran or pyrrole) by means of a C–N bond, comparable with the N-glycosidic bond of nucleosides, in **4**–**6** the purine-like scaffold is instead always connected via a C–C bond, thus increasing the suitability of N atoms to interactions with accessible protein residues. In fact, both the 2H-pyrazole[3-4,d]pyrimdine scaffold of **4** and **5** and the pyrazole[3-4,b]pyridine scaffold of **6** are characterized by N atom groups that can act as either acceptors or donors of H bonds.

**Table 4:** Ligand ID, docking scores and molecular structure of selected hybrid hits

Ligand ID	Docking score XP	Glide ligand efficiency	Structure
4	− 12.098	− 0.417	
5	− 11.024	− 0.394	
6	− 9.783	− 0.466

The higher efficiency of ligands **4** and **5** in doing interactions with the binding site leads to more favorable docking scores than those calculated for either ligand **1**–**3** or other compounds obtained for the diverse library set. Indeed, by analyzing their docking poses (Fig. 6), ligands **4** and **5** not only maintain the salt bridge with the Glu168 and Asp167, but also their interaction into the binding pocket are improved, where the complementarity between ligands and surrounding residues is clearly enhanced. These specific interactions characterized also the binding motives of ligand **6** although its docking score is comparable to those computed for **1** and **2**.

The ligand **4**, predicted to be the best binding compound, is the only selected hybrid molecule with two positively charged groups interacting with the carboxylic side chains of both Asp167 and Glu168. The C6=O carbonyl group establishes a H bond with the backbone N–H of Asp167. The N2–H and N5–H on the pyrazole[3-4,d]pyrimidine scaffold are involved into two H bonds with the backbone C=O moiety of Tyr169 and with the carboxylate of Asp178, respectively. The two OH groups on the tetrahydrofuran ring are also important because they interact with the backbone C=O of Pro165.

Ligand **5**, designed around the scaffold sf-298a as ligand **4**, gives rise to an analogous interactions’ pattern with the binding site, although this ligand bears only one positively charged alkylamine tail. Such a difference may explain the less favorable value of docking score of ligand **5** compared to ligand **4**.

Ligand **6**, obtained by the scaffold sf-128 (1H-pyrazolo[3,4-b]pyridine), establishes three H bonds with backbone moieties belonging to Pro165, Asp167 and Tyr169. As observed also in the docking poses of ligand **4** and **5**, the positively charged tail of ligand **6** interacts with the COO^–^ groups of Asp167 and Glu168.

### MD simulation of E200K-ligand complexes and MEP analysis

The MD simulation of the complexes between PrP-E200K and ligands **1**–**6**, predicted to be the most active binders, were then performed to both assess the stability of each binding hypothesis and to provide for representative structure of bound PrP-E200K to be subjected to further analyses.

RMSD analysis of MD trajectories reveals that all ligands rearrange their binding poses during the simulation. This evidence can be appreciated also looking at the percentage of H bonds occupancy established between ligands and protein (Table S3). With exception of ligand **3**, all binders are characterized by stable trajectory over all the simulation time, with some variation due to fluctuation of C-terminal portion (Figs. S5–S10).

The analysis of minimum distance between selected ligands and protein confirms that ligand **1** remains in the binding pocket during all trajectory (Fig. S11).

On the contrary, both ligand **2** and **3** detach from the binding site at 42 and 54 ns, respectively (Figs. S12 and S13 in SI), although ligand **2** remains anchored to the protein by interacting with other residues of the BR1, and ligand **3** establishes only transient interactions with other protein regions and, around 130 ns, it completely loses any contacts with the protein. In the last 40 ns of trajectory, ligand **3** established again transient contacts with different regions of protein. Based on this outcome, we cannot consider ligand **3** as a potential PrP-E200K binder.

Interestingly, both **2** and **3** were affected by the least favorable XP scores thus suggesting that the docking scores provided a correct assessment of the relative binding stability.

On the other hand, the selected hybrid ligands **4**–**6** remained all stably bound within the pocket BR1_4204 (Figs. S14–S16 in SI), thus corroborating the enhancement of ligand–protein fitness achieved via the hybridization of ligands **1**–**3** with the selected fragments.

The last 50 ns of the production trajectories were analyzed by a clustering procedure and the results are summarized in Table S3: for each PrP-E200K-ligand complex we obtained three or four clusters of which the first is always the most populated one.

The representative structures (middle elements) of the four clusters obtained by the ligand **1** trajectory (Fig. S17) show interactions with backbone, as Asn171 C=O, Pro165 C=O and Asn171 NH, for which the H bond analysis over all the trajectory indicates high values of occupancy, but also evidencing that the salt bridges with negatively charged residues Asp167 and Glu168 are not stable (Table S3). The H bond occupancies for these latter interactions are 11% and 7%, respectively, suggesting that the aliphatic amine group rather interacts with the solvent for the most part of the simulation time. However, the H bonds established with residues into the binding site are strong enough to maintain ligand **1** in the pocket.

On the contrary, ligand **2** leaves the pocket after the break of H bonds with C=O moieties of Tyr169 and Asp178, and this happens rapidly by analyzing the trajectory and confirmed by the extremely low H bonds occupancies of 0.30% and 0.25%, respectively. However, the molecule preserves salt bridges with Asp167 and Glu168 and its aromatic scaffold, although outside the pocket, still interacts with Tyr169 by means of $\pi {-}\pi$ stacking. Moreover, one aliphatic tail interacts with Ser231 C=O group. This particular binding conformation is observed in 3 out of 4 representative structures of clusters (Fig. S18).

As previously mentioned, ligand **3** loses the contact within the pocket (with Asp167 C=O and with Tyr169 C=O) and escapes from its place. The representative structures of clustering reflect this situation (Fig. S19).

So, for both ligands **2** and **3**, the interactions between scaffold and residues within the pocket detected by docking calculations are not stable and they are rapidly broken during MD. That is, the salt bridges between alkyl amine moieties and Asp167 and Glu168 are not adequately strong to maintain **2** and **3** in the pocket.

Thus, considering the three ZINC ligand **1**–**3**, the MD results indicate that the contacts established by N–H function of heterocyclic rings with residues within the binding site are crucial to maintain ligands stable into the pocket, while the positively charged tails, interacting with Asp167 and Glu168, are basically necessary to act as charge-based inhibitors.

The hybrid ligands were designed exactly to enhance the interaction between the N-bicylic ring and binding sites. MD analysis indicate that ligand **4**, the predicted strongest binder, conserves all the interactions of the scaffold 2H-pyrazolo[3,4-d]pyrimin-4-one (sf-298a) with residues inside the pocket (Pro165, Asp167, Tyr169, Asn174 and Asp178), detected by docking calculations. Conversely from docking pose, the pentylamine tail establishes a strong ionic interaction with Asp167 and Glu168 while the shortest one (ethylamine) is oriented to the negatively charged C-terminal region of the protein. Thus, while the pentylamine tail shields the negative charges due to the Asp167 and Glu168, the second tail is able to reach the C-terminus and, dulling the negative charge of this region, increases the efficiency as charge-based inhibitor (Fig. S20). As additional consequence, the C-terminal portion is oriented toward the ligand and for certain time interacts with it (6% of occupancy). This result has been observed only for ligand **4** suggesting that it may have all the main features to act as potential charge-based inhibitors.

Ligand **5** shares the same scaffold with **4**, and also in this case the interactions with C=O moieties of Glu168 and Pro165, and with the side chain of Glu178, are conserved during the simulation, although some of them are not stable (Fig. S21).

On the contrary, ligand **6** is maintained in the pocket only by $\pi - \pi$ interaction with Tyr169; in this case, MD calculations display that the H bonds predicted by docking are in fact not stable and are broken during the simulation (Fig. S22).

In order to investigate the effect of the ligands as charge-based inhibitors of aggregation propensity of PrP-E200K, comparative analyses of both unbound and bound PrP-E200K MEP profiles were performed, via the previously reported computational workflow [17–19].

**Fig. 7:**
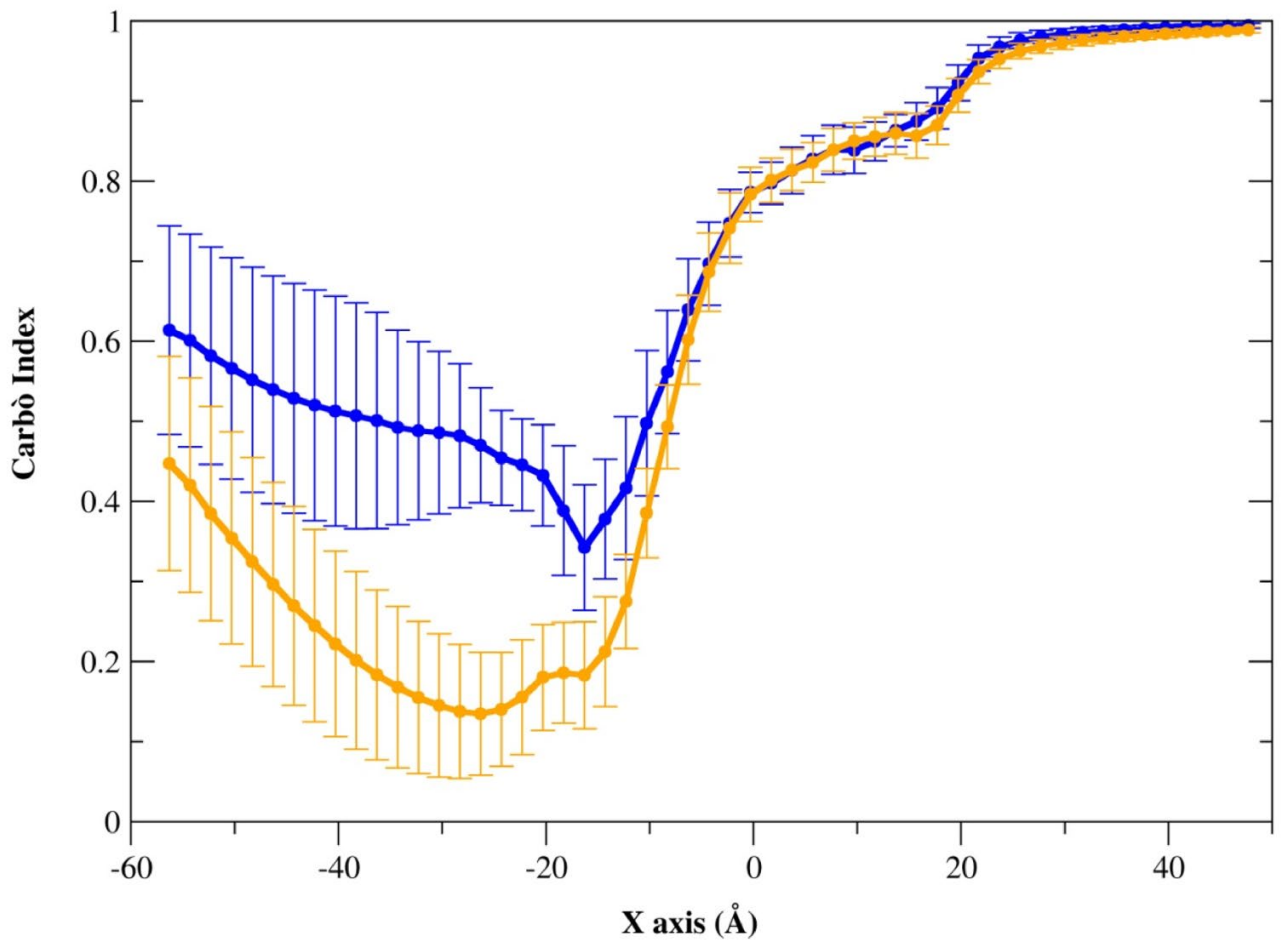
Profiles of MEP similarity of E200K-ligand **1** (blue) and E200K-ligand **4** (orange) versus unbound PrP-E200K

The MEP similarity between unbound E200K and E200K-ligands subsets was then investigated through the calculation of Carbò similarity profiles. As shown in Fig. 7, the two regions of interest were highlighted in the similarity profile comparing ligand **1** and **4**.

In the − 60 to 0 Å segment, delimiting the region of space that includes the binding site BR1_4204, the profile presents a minimum located around − 18 Å, in proximity of the bundle formed by the C-terminal side of H3 and the S2–H2 loop bearing the Asp167 and Glu168. Notably, large standard deviation values characterize the Carbò similarity profile in this region of space, to be probably ascribed to the higher mobility of the charge carried by the ligand in the binding site.

**Fig. 8:**
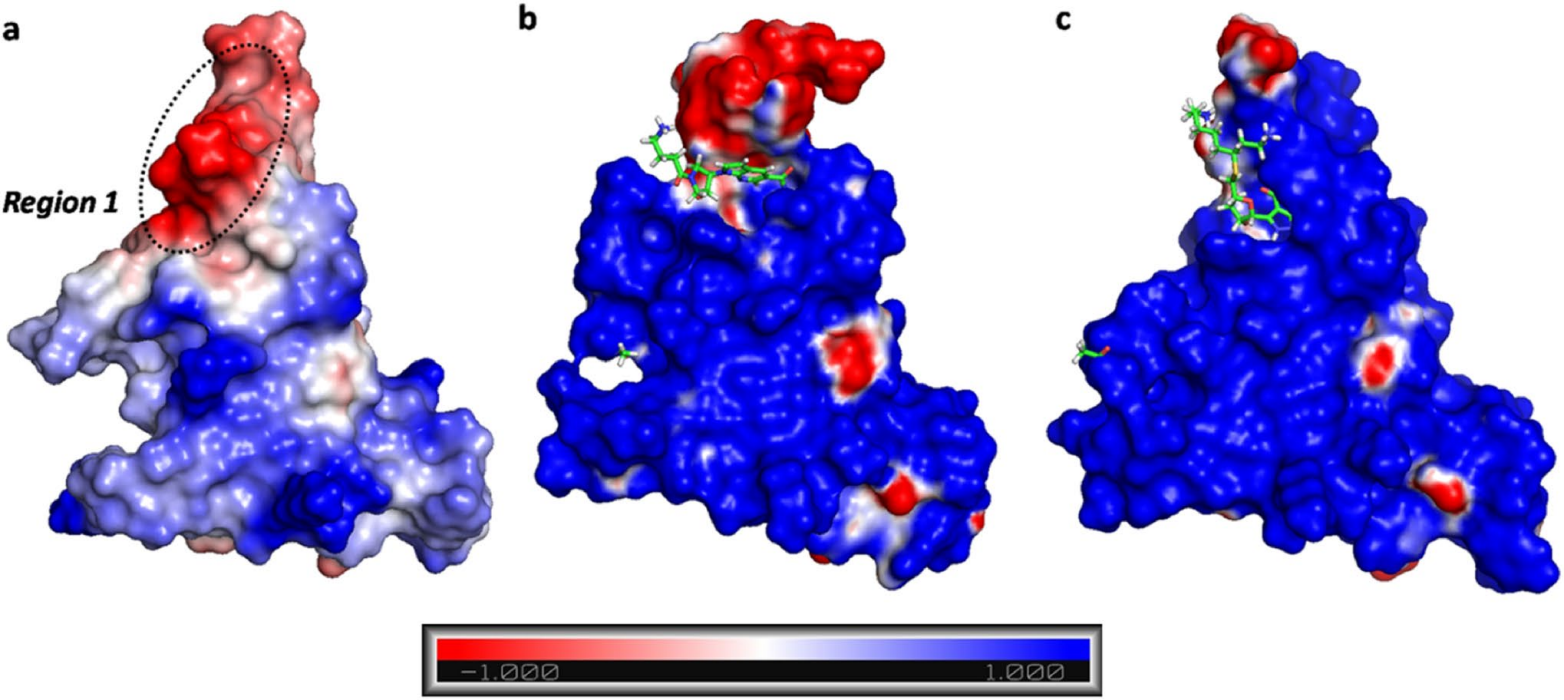
The averaged MEP profiles of **a** PrP-E200K, **b** E200K-ligand **1** and **c** E200K-ligand **4** complexes. The negatively and positively charged regions are reported in red and blue, respectively. MEPs are computed and reported in arbitrary unit ranging in the − 1.0 up to + 1.0

In the second part of the graph (x > 0) the similarity profile steeply increases to reach an asymptotic trend for x > 30. By increasing the distance from the binding pocket, rapidly the Carbò similarity profile tends to the value of one, thus indicating that the MEP of the bound and unbound systems are almost the same in this region of space. Similar evidences were also found in the similarity profiles of the other ligands, with the exception of ligand **3** that does not form a stable adduct with PrP-E200K. As shown in Fig. 7, the best PrP-E200K binders, **1** and **4**, show comparable similarity profiles even though the minimum value was computed for ligand **4**. Such an effect can be even more evidenced by visualizing the MEP isosurfaces on the 3D structure of either unbound PrP-E200K or its protein–ligand complexes (Fig. 8). In the unbound PrP-E200K (Fig. 8a), Region 1 is negatively charged whereas it appears almost completely reverted to be positively charged upon the interaction with ligand **1** (Fig. 8b). Interestingly, the C-terminal region, negatively charged as well, is not significantly affected by such binder. On the other hand, the ligand **4** presents an ethylamine tail that can reach the C-terminal region and shield its negative charge by leading to a lowest minimum in the Carbò similarity around 23 Å (Fig. 8c). The interactions of ligand **4** with the C-terminal region fade the typical fluctuation of this flexible region [17] by leading to smaller standard deviation values compared to the other systems. Based on this result, ligand **4** appears to be more effective in potentially reverting/shielding the negative charges of Region 1, and, eventually, disrupting the Region 1–Region 3 electrostatic interaction between two PrP-E200K units.

The similarity profiles of the other protein–ligand complexes, by the only exception of the complex of ligand **3** (bound at PrP-E200K for a short time as already stated), are characterized by similar features although, compared to ligand **4**, the minimum of similarity ranges at higher values (Figs. S23–S26).

## Discussion

The capability of the E200K mutant of human PrP to early aggregate in the cellular form proposed in previous studies [17–19] has been imputed to the presence of two oppositely charged portions of the folded domain (120–231) of this protein, named Region 1 (negative) and Region 3 (positive). In this structural framework, we hypothesized that charged binders of the PrP-E200K protein could act as disruptors of the Region 1–Region 3 electrostatic complementarity, thus, blocking the amyloid progression of this mutant in the early stages (Fig. 2).

The experimental NMR structure of the 90–231 segment of PrP-E200K [59] was preliminary inspected to unveil the presence of suitable binding sites. However, the scanning of the 30 NMR conformations of E200K (PDB ID 1FO7) did not lead us to detect binding sites with an acceptable druggability profile, probably because the NMR models are usually built to capture the average structure of the system, thus, majorly reflecting the most populated conformation of the protein. On the other hand, poorly populated conformations of the PrP-E200K may be characterized by highly druggable binding sites that could be effectively targeted by molecular binders.

Thus, MD calculations were performed to explore the conformational space of the unbound PrP-E200K and to assess the presence of transient or short-lived pockets accessible to small molecules [70].

Although these computational data had been already reported elsewhere [18], the equilibrated trajectory of the 120–231 segment of PrP-E200K structure was newly analyzed with a twofold aim: (i) the identification of suitable binding sites to be targeted by small drug-like ligands, and (ii) to assess the capability of selected ligands to hamper the electrostatic complementarity between Region 1 and Region 3. Although small molecular binders of the prion protein have been already identified [36, 37], the lack of well-characterized pockets in the structure of PrP-E200K protein led us to prioritize the seeking of potential binding sites. At this purpose, we developed a procedure that operates the automated SiteMap analysis of the protein conformations retrieved from a MD trajectory for the identification of binding sites. This computational tool also performed an evaluation of the druggability profile of each site, identifying the most promising binding pockets per each protein conformation retrieved from the MD trajectory. On the other hand, this tool allows an easy identification of binding regions that can be defined as the protein portions characterized by a high incidence (in the time) of binding sites, i.e., where the binding sites appear more frequently. Such analysis allowed us to identify a binding site featured with a higher druggability profile, named BR1_4204, located within the C-terminal of H2 and H2–S1 loop, proximal to Region 1. Interestingly, BR1 is different from the binding region of chlorpromazine and thiamine in murine and hamster prion proteins, respectively, whose complexes were characterized by NMR and X-ray experiments [71, 72].

The BR1_4204 site is featured by several negative charged residues as Asp167, Glu168 and Asp178, able to act as H bond acceptors or to establish salt bridges with charged ligands. Moreover, it is characterized by the presence of Tyr169 accommodating for $\pi - \pi$ interactions. These non-covalent interactions (hydrogen bonding and charge–charge interactions), as well as the aromatic contacts, are typical of molecules able to bind amyloidogenic proteins, especially of amyloid inhibitors derived from natural products [73–75]. In this view, the interaction with Tyr169 may be significant by considering that it has been proposed how $\pi - \pi$ interactions can hamper the proteins aggregation process and protein misfolding [76], and suggested that aromatic interactions may be crucial in the aggregation process [77]. Moreover, it is worth noting that the Tyr169 is strictly conserved in mammalian PrPs likely implicated in the self-assembly process of E200K monomers through its involvement in $\pi - \pi$ stacking that are crucial for the formation of amyloid core [78–80].

Prusiner et al. highlighted the importance of Glu168 and other residues (as Gln172, Ile215, and Glu219) in prion aggregation process, showing that the stability of the molecular complex between PrP^C^ and partner proteins was related to those specific residues [81]. Based on those outcomes, potential anti-prion scaffolds designed to target those residues were identified in several pyridine dicarbonitriles derivatives, able to inhibit the PrP^Sc^ formation in a dose-dependent manner [82, 83]. Therefore, all those evidences support our outcomes suggesting that molecules able to bind the main residues characterizing BR1_4204 may effectively lead to the anti-prion effect.

**Fig. 9:**
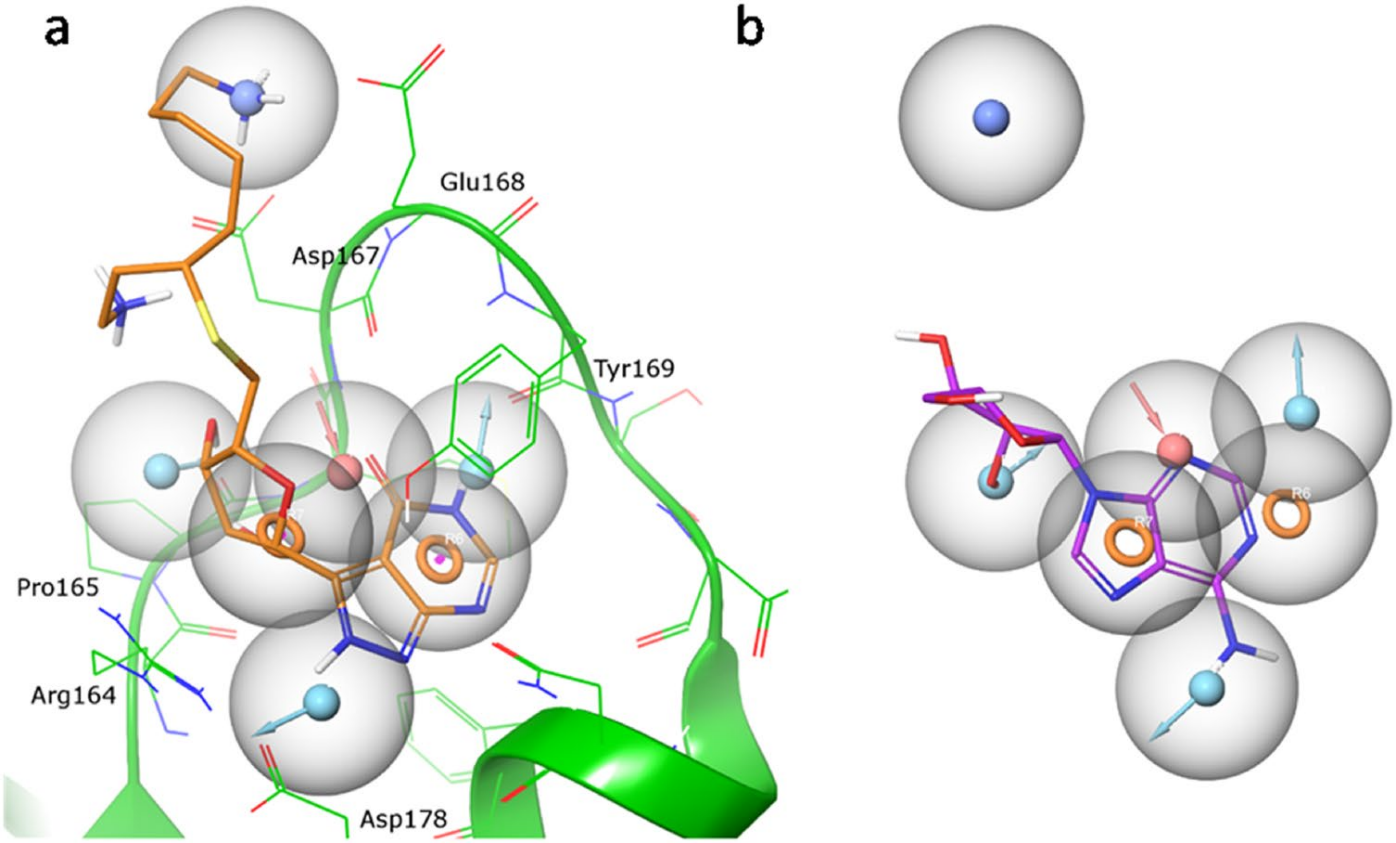
Pharmacophore hypothesis obtained accounting main interactions formed by docked virtual hits in the E200K binding site. **a** Projection of the features in the E200K binding site with docked pose of ligand **4**. **b** Alignment of vidarabine in the pharmacophore hypothesis. Pharmacophore features corresponding to negative charge are represented as a blue sphere, H-bond donor as cyan arrow, H-bond acceptor as red arrows, and the aromatic feature as an orange ring

The characterization of potential binders of the BR1_4204 site was then performed within a two-stage workflow. In the first step, a site-directed docking campaign allowed the identification of three hits and a preliminary evaluation of their fitness. In fact, even the top ranked ligands **1**–**3** evaluated at this stage were characterized by a low or moderate fitness. Thus, in a second step, molecular fragments were docked in the BR1_4204 site and the top ranked fragments were eventually hybridized with ligands **1**–**3**, and the new library submitted to docking to gain ligands **4**–**6**. Such hybridization resulted to be crucial and allowed obtaining ligands with augmented binding features. The subsequent molecular dynamics simulation of the binding complexes of ligands **1**–**6** evidenced that **1**, **4**, **5** and **6** form stable adducts with the PrP-E200K, whereas both **2** and **3** are detached from the BR1_4204 site after 50 ns. This outcome provided a further corroboration to the effectiveness of hybridization. Notably, all ligands **1**–**6** possess a similar structural scheme characterized by a bicyclic aromatic scaffold, a five-membered ring, and one or two hydrocarbon tails terminated by a primary amino group. Therefore, a pharmacophore hypothesis has been generated accounting for the most conserved contacts between docked ligands and the targeted E200K binding site (Fig. 9a), and in particular: H-bond donor to Tyr169 NH, H-bond acceptor to Asp167 CO, two aromatic rings to Tyr169 and Arg164 side chains, H-bond donor to Asp178 carboxylate, H-bond donor to Pro165 CO, and the positive charge between Glu168 and Asp167.

Indeed, the heteroatomic groups decorating either ring scaffolds were found to form hydrogen bonds with several residues of the BR1_4204 binding site. However, our calculations unveiled how the aromatic rings of ligands **1**–**6** are involved in $\pi - \pi$ stacking with the phenol group of Tyr169 that, as above stated, is reputed to play a crucial role in the PrP-E200K aggregation. Therefore, the positively charged amino group of each ligand forms strong hydrogen bond interactions with the carboxylate groups of Asp167 and Glu168; such an interaction is expected to attenuate the negative charge of Region 1, consistently with our postulates. It is also worth noting how in the top ranked ligand **4**, the presence of a second hydrocarbon chain with amino terminal, able to form a strong hydrogen bond interaction with the terminal carboxylate of residue Ser231, leads to both the stabilization of the protein–ligand affinity and the further attenuation of the negative charge. These evidences suggest that the BR1_4204 binding site, identified by our computational approach, can be effectively targeted to disrupt the aggregation properties of Region 1, and, presumably, hamper the prion aggregation at its early stages, as schematized in Fig. 2. Noticeably, several studies have underlined the crucial role played by electrostatic interactions in amyloid formation and that the assembly via charge-charge interaction may be a first step of amyloidogenic process [84–88], thus, disrupting such an interaction can be a possible drug strategy as previously shown for other amyloidogenic proteins [22, 89].

To better assess the effect of ligand binding at BR1_4204 on the electrostatic properties of the PrP-E200K protein, the most representative conformations of the ligands **1**–**6** bound complexes were analyzed in comparison to the representative structures of unbound PrP-E200K. Such a pairwise comparison of protein structures allows a straightforward quali-quantification of the electrostatic similarity between two proteins through the calculation of Carbò index profiles [17, 18]. In this case, the analysis of Carbò index profiles clearly showed how the binding of cationic ligands **1**, **2** and **4**–**6** induces a sensible decrease of similarity in the PrP-E200K portion corresponding to the Region 1. Moreover, the degree of Carbò similarity was found to be sensitive of both the ligand affinity and charge, as shown by the lowest similarity value detected with the ligand **4** complex. This evidence indicates that the MEP analysis presented here can be effectively employed to assess the ability of binders to act as potential charge-based inhibitors of protein–protein interactions [73].

Additional studies are necessary to assess the significance of the BR1_4204 and the real capability of the ligand **4** to hit this site, inducing an anti-prion response. Although our outcomes originated exclusively from theoretical studies, it is worth noting that ligands **1**–**6**, and, in particular, ligand **2**, characterized by 9-(tetrahydrofuran-2-yl)-9H-purin-6-amine scaffold, are structurally comparable to the antiviral agent vidarabine (adenine arabinoside), whose chemical structure is reported in Fig. S27. Interestingly, vidarabine is the unique antiviral drug for which a transient therapeutic, anti-prion response has been reported [90]. Notably, aligning the vidarabine structure to the defined pharmacophore, a quite good matching is obtained with 5 out of 7 sites mapped by ligand functional groups, suggesting that this molecule could bind the identified site (Fig. 9b). This evidence may further support the potential anti-prion activity of ligand **4** exerted by hampering or interfering with the early aggregation of the PrP-E200K protein. In this view, other antiviral drugs with purine scaffold can be considered as good starting point for designing new potential anti-prion drugs.

## Conclusion

In this work, we applied a multistep computational workflow based on MD and molecular docking calculations in order to detect the most promising binding sites on PrP-E200K protein and to identify potential binders with putative anti-prion effect. The scan of MD trajectory allowed us to find a hidden conformation, not detected in the NMR protein structure, of a specific binding region with promising druggable features. The identified binding site, named BR1_4204, is delimited by Arg164, Pro165, Met166, Glu168, Tyr169, Ser170, Asn171, Gln172, Asn174, Phe175, Pro177, and Asp178. In details, Tyr169 and Glu168 are reported to be significant for misfolding process and prion aggregation. We also found, through a combination of high-throughput virtual screening and scaffold hopping approach, six potential E200K binders characterized by aromatic N-bicyclic structure, and with one or two positive charges. According to our computational prevision, the predicted most active compound, ligand **4**, not only hits Asp168 and Tyr169 but also dulls the negative charges of Region 1 and of C-terminal region. This outcome corroborates that ligand **4** may effectively act as charge-based inhibitor hampering the early state of PrP^C^-E200K aggregation, according to our model. Moreover, the selected virtual hits share many structural similarities with vidarabin, the only antiviral drug that showed some therapeutic effect against prion disease, thus, further consolidating the potential anti-prion activity of ligand **4**. Additional investigations and, more specifically, experimental studies are required to verify our computational outcomes and the effectiveness of ligand **4** as anti-prion compound.

## Supplementary Information

Below is the link to the electronic supplementary material.

Supplementary file1 (PDF 2552 KB)

## Abbreviations

BR: Binding region
CJD: Creutzfeldt–Jakob disease
E200K: Mutant of prion protein where Glu200 is muted in Lys200
FMO: Fragment molecular orbital method
HTVS: High-throughput virtual screening
MEP: Molecular electrostatic potential
MD: Molecular dynamics
MIF: Molecular interaction field
NMR: Nuclear magnetic resonance
PDB: Protein Data Bank
PrP: Prion protein
PrP^C^: Cellular prion protein
PrP^Sc^: Scrapie prion protein
sf: Schrodinger fragment
SP: Standard precision docking protocol implemented in Glide (Schrodinger suite)
wt: Wild type
XP: Extra precision docking protocol implemented in Glide (Schrodinger suite)

## References

[1] Prusiner SB (1982). Novel proteinaceous infectious particles cause scrapie. Science.

[2] Prusiner SB (1998). Prions. PNAS.

[3] Eraña H (2019). The Prion 2018 round tables (II): Aβ, tau, α-synuclein… are they prions, prion-like proteins, or what?. Prion.

[4] Watts JC, Prusiner SB (2018). β-amyloid prions and the pathobiology of Alzheimer’s disease. Cold Spring Harb Perspect Med.

[5] Johnson NR, Condello C, Guan S, Oehler A, Becker J, Gavidia M, Carlson GA, Giles K, Prusiner SB (2017). Evidence for sortilin modulating regional accumulation of human τ prions in transgenic mice. PNAS.

[6] Woerman AL, Kazmi SA, Patel S, Aoyagi A, Oehler A, Widjaja K, Mordes DA, Olson SH, Prusiner SB (2017). Familial Parkinson’s point mutation abolishes multiple system atrophy prion replication. PNAS.

[7] Prusiner SB, Woerman AL, Mordes DA, Watts JC, Rampersaud R, Berry DB, Patel S, Oehler A, Lowe JK, Kravitz SN, Geschwind DH, Glidden DV, Halliday GM, Middleton LT, Gentleman SM, Grinberg LT, Giles K (2015). Evidence for α-synuclein prions causing multiple system atrophy in humans with parkinsonism. PNAS.

[8] Chiti F, Dobson CM (2006). Annu Rev Biochem.

[9] Zahn R, Liu A, Luhrs T, Riek R, von Schroetter C, Garcia FL, Billeter M, Calzolai L, Wider G, Wüthrich K (2000). NMR solution structure of the human prion protein. PNAS.

[10] Simon ES, Kahana E, Chapman J, Treves TA, Gabizon R, Rosenmann H, Zilber N, Korczyn AD (2000). Creutzfeldt–Jakob disease profile in patients homozygous for the PRNP E200K mutation. Ann Neurol.

[11] Machado CF, Beraldo FH, Santos TG, Bourgeon D, Landemberger MC, Roffé M, Martins VR (2012). Disease-associated mutations in the prion protein impair laminin-induced process outgrowth and survival. J Biol Chem.

[12] Nuytemans K, Theuns J, Cruts M, Van Broeckhoven C (2010). Genetic etiology of Parkinson disease associated with mutations in the SNCA, PARK2, PINK1, PARK7, and LRRK2 genes: a mutation update. Hum Mutat.

[13] Goedert M, Jakes R (2005). Mutations causing neurodegenerative tauopathies. Biochem Biophys Acta.

[14] Rosenmann H, Talmor G, Halimi M, Yanai A, Gabizon R, Meiner Z (2001). Prion protein with an E200K mutation displays properties similar to those of the cellular isoform PrP(C). J Neurochem.

[15] Zhang Y, Swietnicki W, Zagorski MG, Surewicz WK, Sönnichsen FD (2000). Solution structure of the E200K variant of human prion protein implications for the mechanism of pathogenesis in familial prion diseases. J Biol Chem.

[16] Sorrentino S, Bucciarelli T, Corsaro A, Tosatto A, Thellung S, Villa V, Schininà ME, Maras B, Galeno R, Scotti L, Creati F, Marrone A, Re N, Aceto A, Florio T, Mazzanti M (2012). Calcium binding promotes prion protein fragment 90–231 conformational change toward a membrane destabilizing and cytotoxic structure. PLoS ONE.

[17] Storchi L, Paciotti R, Re N, Marrone A (2015). Investigation of the molecular similarity in closely related protein systems: the PrP case study. Proteins.

[18] Marrone A, Re N, Storchi L (2016). The effects of Ca^2+^ concentration and E200K mutation on the aggregation propensity of PrP^C^: a computational study. PLoS ONE.

[19] Paciotti R, Storchi L, Marrone A (2019). An insight of early PrP-E200K aggregation by combined molecular dynamics/fragment molecular orbital approaches. Proteins.

[20] Sang JC, Lee J, Dear AJ, De S, Meisl G, Thackray AM, Bujdoso R, Knowles TPJ, Klenerman D (2019). Direct observation of prion protein oligomer formation reveals an aggregation mechanism with multiple conformationally distinct species. Chem Sci.

[21] Dear AJ, Meisl G, Šarić A, Michaels TCT, Kjaergaard M, Linse S, Knowles TPJ (2020). Identification of on- and off-pathway oligomers in amyloid fibril formation. Chem Sci.

[22] Patil SM, Alexandrescu AT (2015). Charge-based inhibitors of amylin fibrillization and toxicity. J Diabetes Res.

[23] Sim VL (2012). Prion disease: chemotherapeutic strategies. Infect Disorders.

[24] Caughey B, Race RE (1992). Potent inhibition of scrapie-associated PrP accumulation by Congo red. J Neurochem.

[25] Woody AM, Reisbig RR, Woody RW (1981). Spectroscopic studies of Congo red binding to RNA polymerase. Biochim Biophys Acta.

[26] Ladogana A, Casaccia P, Ingrosso L, Cibati M, Salvatore M, Xi YG, Masullo C, Pocchiari M (1992). Sulphate polyanions prolong the incubation period of scrapie-infected hamsters. J Gen Virol.

[27] Gilch S, Winklhofer KF, Groschup MH, Nunziante M, Lucassen R, Spielhaupter C, Muranyi W, Riesner D, Tatzelt J, Schatzl HM (2001). Intracellular re-routing of prion protein prevents propagation of PrP(Sc) and delays onset of prion disease. EMBO J.

[28] Caughey B, Raymond LD, Raymond GJ, Maxson L, Silveira J, Baron GS (2003). Inhibition of protease-resistant prion protein accumulation in vitro by curcumin. J Virol.

[29] Hafner-Bratkovic I, Gaspersic J, Smid LM, Bresjanac M, Jerala R (2008). Curcumin binds to the alpha-helical intermediate and to the amyloid form of prion protein—a new mechanism for the inhibition of PrP(Sc) accumulation. J Neurochem.

[30] Riemer C, Burwinkel M, Schwarz A, Gultner S, Mok SW, Heise I, Holtkamp N, Baier M (2008). Evaluation of drugs for treatment of prion infections of the central nervous system. J Gen Virol.

[31] Hnasko R, Bruederle CE (2009). Inoculation of scrapie with the self-assembling RADA-peptide disrupts prion accumulation and extends hamster survival. PLoS ONE.

[32] Doh-Ura K, Iwaki T, Caughey B (2000). Lysosomotropic agents and cysteine protease inhibitors inhibit scrapie-associated prion protein accumulation. J Virol.

[33] Korth C, May BC, Cohen FE, Prusiner SB (2001). Acridine and phenothiazine derivatives as pharmacotherapeutics for prion disease. PNAS.

[34] Ryou C, Legname G, Peretz D, Craig JC, Baldwin MA, Prusiner SB (2003). Differential inhibition of prion propagation by enantiomers of quinacrine. Lab Invest.

[35] Kocisko DA, Caughey B (2006). Mefloquine, an antimalaria drug with antiprion activity in vitro, lacks activity in vivo. J Virol.

[36] Barreca ML, Iraci N, Biggi S, Cecchetti V, Biasini E (2018). Pharmacological agents targeting the cellular prion protein. Pathogens.

[37] Astolfi A, Spagnolli G, Biasini E, Barreca ML (2020). The compelling demand for an effective PrP^C^-directed therapy against prion diseases. ACS Med Chem Lett.

[38] Yamaguchi K, Kamatari YO, Ono F, Shibata H, Fuse T, Elhelaly AE, Fukuoka M, Kimura T, Hosokawa-Muto J, Ishikawa T, Tobiume M, Takeuchi Y, Matsuyama Y, Ishibashi D, Nishida N, Kuwata K (2019). A designer molecular chaperone against transmissible spongiform encephalopathy slows disease progression in mice and macaques. Nat Biomed Eng.

[39] Kuwata K, Nishida N, Matsumoto T, Kamatari YO, Hosokawa-Muto J, Kodama K, Nakamura HK, Kimura K, Kawasaki M, Takakura Y, Shirabe S, Takata J, Kataoka Y, Katamine S (2007). Hot spots in prion protein for pathogenic conversion. PNAS.

[40] Biasini E (2019). A designer chaperone against prion diseases. Nat Biomed Eng.

[41] Ishibashi D, Nakagaki T, Ishikawa T, Atarashi R, Watanabe K, Cruz FA, Hamada T, Nishida N (2016). Structure-based drug discovery for prion disease using a novel binding simulation. EBioMedicine.

[42] Van Der Spoel D, Lindahl E, Hess B, Groenhof G, Mark AE, Berendsen HJ (2005). Fast parallel algorithms for short-range molecular dynamics. J Comput Chem.

[43] Berendsen HJC, Postma JPM, Van Gunsteren WF, Hermans J (1981). Intermolecular forces.

[44] Jorgensen WL, Maxwell DS, Tirado-Rives J (1996). Development and testing of the OPLS all-atom force field on conformational energetics and properties of organic liquids. J Am Chem Soc.

[45] Kaminski GA, Friesner RA, Tirado-Rives J, Jorgensen WJ (2001). Evaluation and reparametrization of the OPLS-AA force field for proteins via comparison with accurate quantum chemical calculations on peptides. J Phys Chem B.

[46] Berendsen HJC, Postma JPM, Van Gunsteren WF, Di Nola A, Haak JR (1984). Molecular dynamics with coupling to an external bath. J Chem Phys.

[47] Hess B, Bekker H, Berendsen HJC, Frajie JCEM (1997). LINCS: a linear constraint solver for molecular simulations. J Comput Chem.

[48] Darden TA, York DM, Pedersen LG (1993). Particle mesh Ewald: An N⋅ log (N) method for Ewald sums in large systems. J Chem Phys.

[49] Da Silva AWS, Vranken WF (2012). ACPYPE - AnteChamber PYthon Parser interface. BMC Res Notes.

[50] Bernardi A, Faller R, Reith D, Kirschner KN (2019). ACPYPE update for nonuniform 1–4 scale factors: conversion of the GLYCAM06 force field from AMBER to GROMACS. Software X.

[51] Wang J, Wang W, Kollman PA, Case DA (2006). Automatic atom type and bond type perception in molecular mechanical calculations. J Mol Graph Model.

[52] Wang J, Wolf RM, Caldwell JW, Kollman PA, Case DA (2004). Development and testing of a general AMBER force field. J Comput Chem.

[53] Humphrey W, Dalke A, Schulten K (1996). VMD: visual molecular dynamics. J Mol Graphics.

[54] Schrödinger LLC (2013) Maestro, Version 9.4. New York, NY, USA

[55] Daura X, Gademann K, Jaun B, Seebach D, Van Gunsteren WF, Mark AE (1999). Peptide folding: when simulation meets experiment. Angew Chem Int Ed.

[56] Schrödinger LLC (2010) MacroModel, version 9.8, New York, NY

[57] Still WC, Tempczyk A, Hawley RC, Hendrickson T (1990). Semianalytical treatment of solvation for molecular mechanics and dynamics. J Am Chem Soc.

[58] Di Qiu M, Shenkin P, Hollinger F, Still W (1997). The GB/SA continuum model for solvation. A fast analytical method for the calculation of approximate Born radii. J Phys Chem A.

[59] Zhang Y, Swietnicki W, Zagorski MG, Surewicz WK, Sonnichsen FD (2000). Solution structure of the E200K variant of human prion protein. Implications for the mechanism of pathogenesis in familial prion diseases. J Biol Chem.

[60] Schrödinger LLC. Schrödinger Suite 2017–3: MacroModel, Glide, SiteMap, LigPrep, Maestro; Schrödinger LLC: New York, NY, USA, 2017

[61] Halgren T (2007). New method for fast and accurate binding-site identification and analysis. Chem Biol Drug Des.

[62] Storchi L, SiteMap based binding sites search github repo: https://github.com/lstorchi/scantrj

[63] Blomley R, Weinmann M, Leitloff J, Jutzi B (2014). Shape distribution features for point cloud analysis-a geometric histogram approach on multiple scales. ISPRS Ann Photogram Remote Sens Spatial Inf Sci.

[64] SiteMap User Manual Copyright © 2015 Schrödinger, LLC

[65] Stank A, Kokh DB, Fuller JC, Wade RC (2016). Protein binding pocket dynamics. Acc Chem Res.

[66] Sterling T, Irwin JJ (2015). ZINC 15—Ligand discovery for everyone. J Chem Inf Model.

[67] Berthold MR, Cebron N, Dill F, Gabriel TR, Kötter T, Meinl T, Ohl P, Thiel K, Wiswedel B (2009). KNIME - the Konstanz information miner: version 2.0 and beyond. Assoc Comput Mach.

[68] Bach S, Talarek N, Andrieu T, Vierfond J, Mettey Y, Galons H, Dormont D, Meijer L, Cullin C, Blondel M (2003). Isolation of drugs active against mammalian prions using a yeast-based screening assay. Nat Biotechnol.

[69] Bajorath J (2017). Computational scaffold hopping: cornerstone for the future of drug design?. Future Med Chem.

[70] Kuzmanic A, Bowman GR, Juarez-Jimenez J, Michel J, Gervasio FL (2020). Investigating cryptic binding sites by molecular dynamics simulations. Acc Chem Res.

[71] Baral PK, Swayampakula M, Rout MK, Kav NN, Spyracopoulos L, Aguzzi A, James MN (2014). Structural basis of prion inhibition by phenothiazine compounds. Structure.

[72] Perez-Pineiro R, Bjorndahl TC, Berjanskii MV, Hau D, Li L, Huang A, Lee R, Gibbs E, Ladner C, Dong YW, Abera A, Cashman NR, Wishart DS (2011). The prion protein binds thiamine. FEBS J.

[73] Ma L, Yang C, Zheng J, Chen Y, Xiao Y, Huang K (2020). Non-polyphenolic natural inhibitors of amyloid aggregation. Eur J Med Chem.

[74] Velander P, Wu L, Henderson F, Zhang S, Bevan DR, Xu B (2017). Natural product-based amyloid inhibitors. Biochem Pharmacol.

[75] Kocisko DA, Baron GS, Rubenstein R, Chen J, Kuizon S, Caughey B (2003). New inhibitors of scrapie-associated prion protein formation in a library of 2000 drugs and natural products. J Virol.

[76] Porat Y, Abramowitz A, Gazit E (2006). Inhibition of amyloid fibril formation by polyphenols: structural similarity and aromatic interactions as a common inhibition mechanism. Chem Biol Drug Des.

[77] Wen LL, Lyu M, Xiao HS, Lan HR, Zuo ZL, Yin ZN (2018). Protein aggregation and performance optimization based on microconformational changes of aromatic hydrophobic regions. Mol Pharm.

[78] Huang D, Caflisch A (2015). The roles of the conserved tyrosine in the β2–α2 loop of the prion protein. Prion.

[79] Huang D, Caflisch A (2015). Evolutionary conserved Tyr169 stabilizes the β2–α2 loop of the prion protein. J Am Chem Soc.

[80] Gallagher-Jones M, Glynn C, Boyer DR, Martynowycz MW, Hernandez E, Miao J, Zee C, Novikova IV, Goldschmidt L, McFarlane HT, Helguera GF, Evans JE, Sawaya MR, Cascio D, Eisenberg DS, Gonen T, Rodriguez JA (2018). Sub-ångström cryo-EM structure of a prion protofibril reveals a polar clasp. Nat Struct Mol Biol.

[81] Kaneko K, Zulianello L, Scott M, Cooper CM, Wallace AC, James TL, Cohen FE, Prusiner SB (1997). Evidence for protein Xbinding to a discontinuous epitope on the cellular prion protein duringscrapie prion propagation. PNAS.

[82] Reddy TR, Mutter R, Heal W, Guo K, Gillet VJ, Pratt S, Chen B (2006). Library design, synthesis, and screening: pyridine dicarbonitriles as potential prion disease therapeutics. J Med Chem.

[83] Guo K, Mutter R, Heal W, Reddy TR, Cope H, Pratt S, Thompson MJ, Chen B (2008). Synthesis and evaluation of a focused library of pyridine dicarbonitriles against prion disease. Eur J Med Chem.

[84] Greenbaum EA, Graves CL, Mishizen-Eberz AJ, Lupoli MA, Lynch DR, Englander SW, Axelsen PH, Giasson BI (2005). The E46K mutation in alpha-synuclein increases amyloid fibril formation. J Biol Chem.

[85] Brender JR, Hartman K, Reid KR, Kennedy RT, Ramamoorthy A (2008). A single mutation in the nonamyloidogenic region of islet amyloid polypeptide greatly reduces toxicity. Biochemistry.

[86] Jones EM, Dubey M, Camp PJ, Vernon BC, Biernat J, Mandelkow E, Majewski J, Chi EY (2012). Interaction of tau protein with model lipid membranes induces tau structural compaction and membrane disruption. Biochemistry.

[87] Zhang M, Ren B, Liu Y, Liang G, Sun Y, Xu L, Zheng J (2017). Membrane interactions of hIAPP monomer and oligomer with lipid membranes by molecular dynamics simulations. ACS Chem Neurosci.

[88] Holmes BB, DeVos SL, Kfoury N, Li M, Jacks R, Yanamandra K, Ouidja MO, Brodsky FM, Marasa J, Bagchi DP, Kotzbauer PT, Miller TM, Papy-Garcia D, Diamond MI (2013). Heparan sulfate proteoglycans mediate internalization and propagation of specific proteopathic seeds. PNAS.

[89] Palhano FL, Lee J, Grimster NP, Kelly JW (2013). Toward the molecular mechanism(s) by which EGCG treatment remodels mature amyloid fibrils. J Am Chem Soc.

[90] Furlow TW, Whitley RJ, Wilmes FJ (1982). Repeated suppression of Creutzfeldt–Jakob disease with vidarabine. Lancet.

