# Supplementary material for: Hampering the early aggregation of PrP-E200K protein by charge-based inhibitors: a computational study
Source: J Comput Aided Mol Des. 2021 Jun 10;35(6):751–70. doi: 10.1007/s10822-021-00393-7 (PMC8213589; doi:10.1007/s10822-021-00393-7)
Supplement: Supplementary file 1 — Supplementary file1 (PDF 2552 KB) [file 10822_2021_393_MOESM1_ESM.pdf]

Supporting Information

Hampering the early aggregation of PrP-E200K protein by charge-based inhibitors: a computational study.

Mariangela Agamennone[1], Loriano Storchi,[1,2] Alessandro Marrone[1], Roberto Paciotti[1*]

[1] Università "G d'Annunzio" di Chieti-Pescara, Department of Pharmacy, Chieti, Italy.

[2] Molecular Discovery Limited, Middlesex, London, United Kingdom.

*** Correspondence to**: Roberto Paciotti (E-mail: r.paciotti@unich.it)

Table of Contenets

Fig. S1 3
Fig. S2 3
Table S1 4
Fig. S3 5
Table S2 6
Fig. S4 7
Table S3 8
Fig. S5 9
Fig. S6 9
Fig. S7 10
Fig. S8 10
Fig. S9 11
Fig. S10 11
Fig. S11 12
Fig. S12 12
Fig. S13 13
Fig. S14 13
Fig. S15 14
Fig. S16 14
Table S4 15
Fig. S17 16
Fig. S18 17
Fig. S19 18
Fig. S20 19
Fig. S21 20
Fig. S22 21
Fig. S23 22
Fig. S24 23
Fig. S25 24
Fig. S26 25
Fig. S27 26

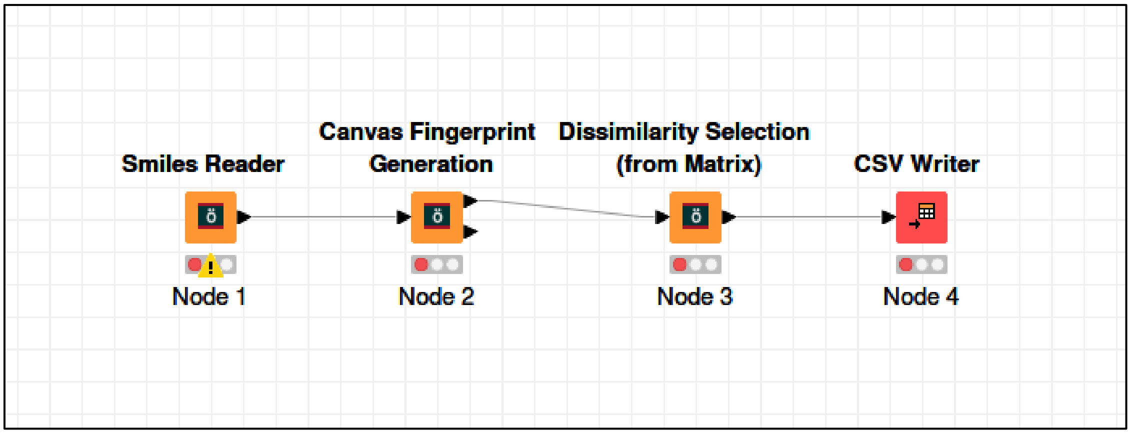

Fig. S1 Knime workflow for diverse library selection

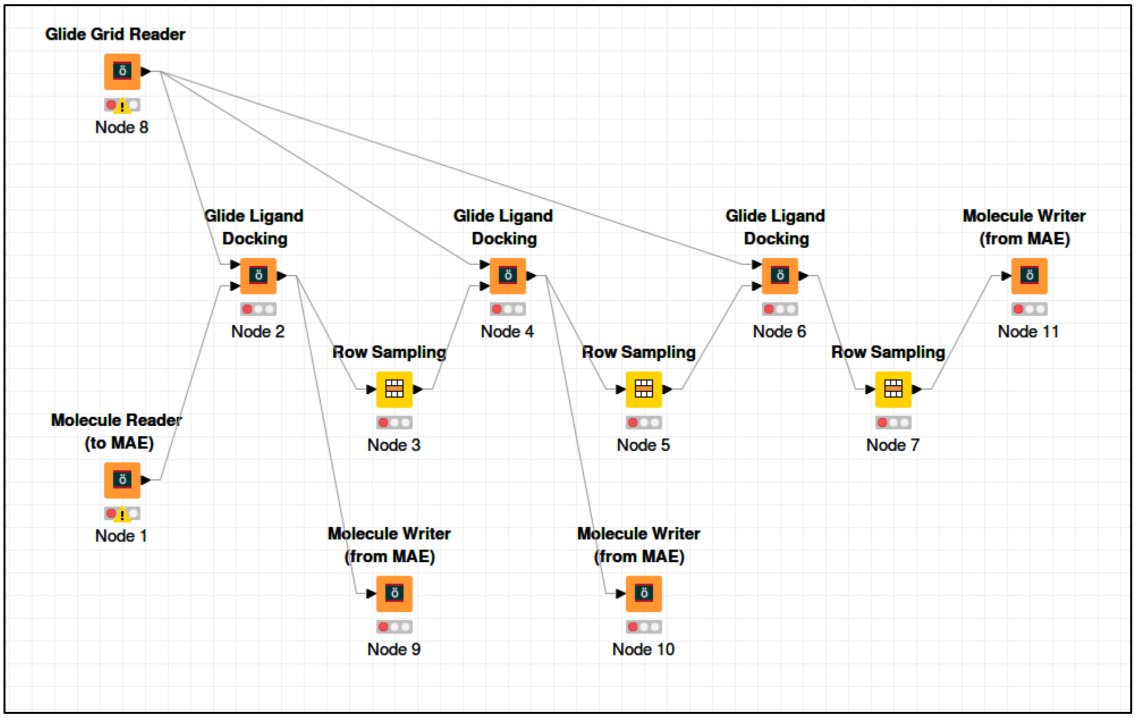

Fig. S2 Knime workflow for the virtual screening protocol

Table S1 SiteMap score values calculated for the top 15 sites identified on the 30 conformations from the NMR structure 1FO7

**Binding Site ID**	**SiteMap score values**										
	**SiteScore**	**size**	**Dscore**	**volume**	**exposure**	**enclosure**	**contact**	**phobic**	**philic**	**balance**	**don/acc**
1FO7_e_BR1	0.909	118	1.02	115.119	0.726	0.41	0.431	0.525	0.509	1.032	0.677
1FO7_f_BR1	0.924	124	1.048	105.858	0.742	0.402	0.392	0.517	0.408	1.27	2.48
1FO7_l_BR1a	0.907	124	1.028	78.933	0.715	0.386	0.374	0.394	0.442	0.893	1.468
1FO7_a_BR1a	0.908	180	1.023	145.646	0.751	0.399	0.39	0.275	0.479	0.574	1.196
1FO7_c_BR2	0.918	140	1.042	95.354	0.79	0.394	0.34	0.224	0.411	0.543	1.724
1FO7_t_BR1a	0.905	131	1.034	70.229	0.787	0.369	0.318	0.27	0.391	0.69	1.073
1FO7_r_BR1	0.883	164	0.989	122.237	0.696	0.386	0.4	0.204	0.561	0.364	1.043
1FO7_i_BR1a	0.907	124	1.04	64.698	0.803	0.365	0.319	0.368	0.366	1.006	1.674
1FO7_l_BR1	0.878	106	0.984	91.581	0.748	0.376	0.379	0.266	0.557	0.478	1.435
1FO7_d_BR1	0.88	102	0.997	72.03	0.765	0.361	0.364	0.256	0.49	0.521	1.307
1FO7_D_BR1a	0.898	138	1.026	84.85	0.795	0.36	0.315	0.236	0.397	0.593	1.032
1FO7_z_BR1a	0.904	109	1.04	75.246	0.797	0.354	0.313	0.266	0.345	0.773	0.702
1FO7_s_BR1	0.866	88	0.967	92.91	0.743	0.406	0.423	0.362	0.485	0.746	1.423
1FO7_u_BR2	0.894	180	1.018	132.441	0.81	0.362	0.333	0.208	0.427	0.486	1.925
1FO7_q_BR1a	0.91	106	1.048	57.753	0.836	0.357	0.289	0.33	0.324	1.018	1.556

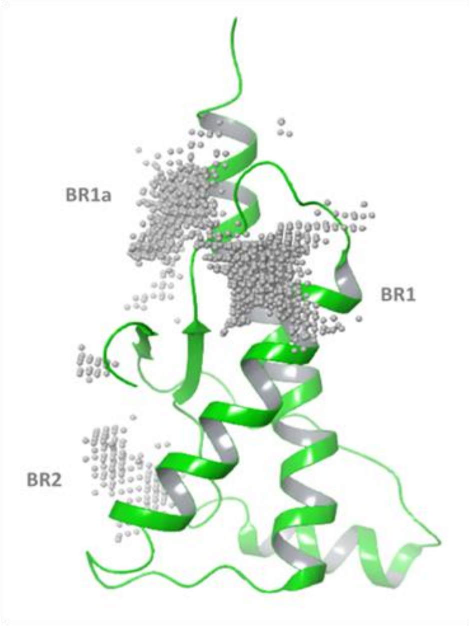

Fig. S3 Best identified binding regions (BRs) on the surface of 1FO7 NMR structures represented as green cartoon. Site points calculated by SiteMap to locate the binding site are represented as grey spheres

Table S2 SiteMap score values calculated for the top 36 sites from the MD trajectory analysis.

**Binding Site ID**	**SiteMap score values**										
	**score**	**size**	**dscore**	**volume**	**exposure**	**enclosure**	**balance**	**linearity**	**planarity**	**sphericity**	**anisotropy**
BR1_4530	0.940	73.0	0.905	127.467	0.411	0.767	1.189	0.257	0.404	0.339	0.661
BR1_8781	0.931	71.0	0.869	133.599	0.360	0.767	1.109	0.277	0.404	0.319	0.681
BR2_4946	0.918	67.0	0.829	119.493	0.391	0.775	1.007	0.306	0.350	0.344	0.656
BR1_5530	0.916	66.0	0.822	138.572	0.353	0.778	0.834	0.203	0.497	0.300	0.700
**BR1_4204**	**0.876**	**57.0**	**0.762**	**114.133**	**0.345**	**0.781**	**0.715**	**0.111**	**0.542**	**0.347**	**0.653**
BR1_3379	0.894	62.0	0.808	127.467	0.421	0.773	0.779	0.139	0.370	0.491	0.509
BR1_5863	0.917	69.0	0.923	140.887	0.444	0.713	1.188	0.117	0.344	0.539	0.461
BR1_4248	0.902	66.0	0.821	132.184	0.405	0.757	0.782	0.252	0.414	0.334	0.666
BR2_2272	0.871	61.0	0.806	112.333	0.435	0.746	1.139	0.221	0.343	0.436	0.564
BR1_5471	0.875	58.0	0.787	106.459	0.448	0.772	0.951	0.184	0.448	0.368	0.632
BR1_4073	0.898	63.0	0.811	119.107	0.417	0.771	0.734	0.243	0.347	0.410	0.590
BR1_3370	0.895	64.0	0.824	130.983	0.434	0.760	0.782	0.112	0.373	0.515	0.485
BR2_4038	0.827	48.0	0.740	87.165	0.448	0.777	1.071	0.259	0.431	0.310	0.690
BR1_5810	0.849	55.0	0.764	104.272	0.421	0.755	1.029	0.155	0.405	0.440	0.560
BR1_4065	0.834	52.0	0.754	108.045	0.395	0.755	0.898	0.226	0.326	0.448	0.552
BR1_9010	0.893	67.0	0.887	135.399	0.477	0.710	1.265	0.201	0.339	0.460	0.540
BR1_3205	0.863	57.0	0.751	125.667	0.436	0.762	0.759	0.150	0.503	0.348	0.652
BR1_3173	0.830	52.0	0.703	89.823	0.447	0.750	0.963	0.204	0.361	0.436	0.564
BR1_3729	0.894	63.0	0.791	122.451	0.452	0.765	0.703	0.326	0.360	0.314	0.686
BR1_5451	0.839	57.0	0.805	131.712	0.467	0.726	1.106	0.336	0.376	0.288	0.712
BR1_3053	0.831	52.0	0.791	101.271	0.480	0.747	1.216	0.242	0.409	0.349	0.651
BR1_4133	0.842	56.0	0.794	95.225	0.440	0.737	0.838	0.050	0.386	0.564	0.436
BR2_3958	0.820	46.0	0.700	82.363	0.477	0.781	0.941	0.264	0.445	0.291	0.709
BR1_3135	0.877	64.0	0.878	132.141	0.475	0.686	1.092	0.180	0.433	0.388	0.612
BR1_4627	0.855	60.0	0.800	121.508	0.474	0.728	0.952	0.354	0.323	0.323	0.677
BR1_4080	0.826	49.0	0.715	91.753	0.467	0.767	0.861	0.317	0.336	0.347	0.653
BR1_4169	0.819	50.0	0.734	87.294	0.405	0.749	0.781	0.197	0.421	0.382	0.618
BR1_3235	0.830	49.0	0.714	93.210	0.473	0.773	0.775	0.281	0.376	0.343	0.657
BR1_2424	0.882	66.0	0.860	137.286	0.480	0.727	0.872	0.186	0.412	0.403	0.597
BR1_5977	0.820	55.0	0.783	97.927	0.455	0.712	1.033	0.206	0.415	0.378	0.622
BR1_4544	0.821	57.0	0.798	105.558	0.477	0.683	1.190	0.244	0.428	0.328	0.672
BR1_8613	0.852	63.0	0.803	113.019	0.471	0.703	0.761	0.264	0.332	0.404	0.596
BR1_4174	0.821	51.0	0.698	93.982	0.452	0.744	0.732	0.304	0.352	0.343	0.657
BR1_2673	0.845	59.0	0.794	118.206	0.478	0.720	0.750	0.300	0.349	0.351	0.649
BR2_4927	0.801	57.0	0.762	95.911	0.467	0.669	0.874	0.348	0.330	0.322	0.678
BR1_3242	0.804	53.0	0.717	105.344	0.518	0.703	0.414	0.253	0.462	0.284	0.716

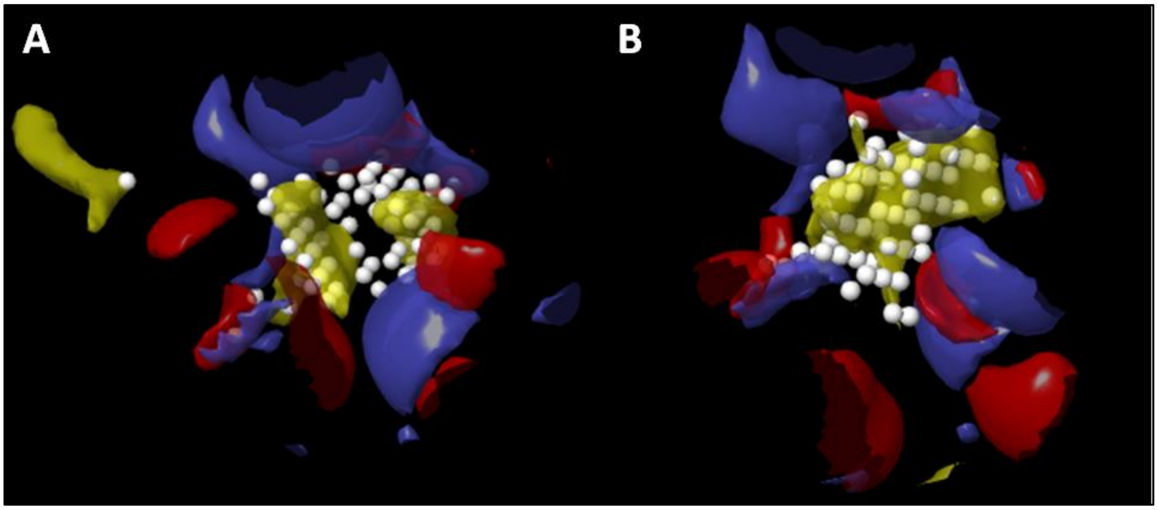

Fig. S4 MIFs calculated for the best site of BR1, obtained from the MD trajectory analysis: a) the site on conformation BR1_4530 reported as an example and b) on conformation BR1_4204, the selected binding site

Table S3 Binding properties of the candidate binders of the E200K-PrP protein

Ligands	Docking score	Resident time	Hydrogen bonds description
**1**	-10.5	200 ns #	Asn171[a](60%-main), Pro165[a] (74% main), Asp167[a] (11%-side), Asn171[d](11%-main), Asn174[a](14-side), Glu168[a](7%-side)
**2**	-7.4	54 ns	Glu168[a] (29% side), Asp167[a](37% side), Ser231[a] (8% side), Met116[a] (4% main)
**3**	-7.0	42 ns	Glu168[a] (24% side), Asp167[a] (5% side)
**4**	-12.1	200 ns #	Asp167[a] (64% side), Glu168[a] (69% side), Tyr169[a] (29% main), Asp178[a] (68% side), Ser231[a] (6% side), Asp167[d] (20% main), Asn174[d] (17% side)
**5**	-11.0	200 ns #	Asp178[a] (34% side), Asp167[a] (40% side), Pro165[a] (69% main), Glu168[a] (7% side), Asp167[d] (6% main)
**6**	-9.8	200 ns #	Asp167[a] (55% side)Glu168[a] (21% side) Asp178[a] (14% side)

[#]binding pose stable over the entire trajectory; [a]H bond acceptor; [d]H bond donor; main: NH or C=O of backbone; chain: sidechain.

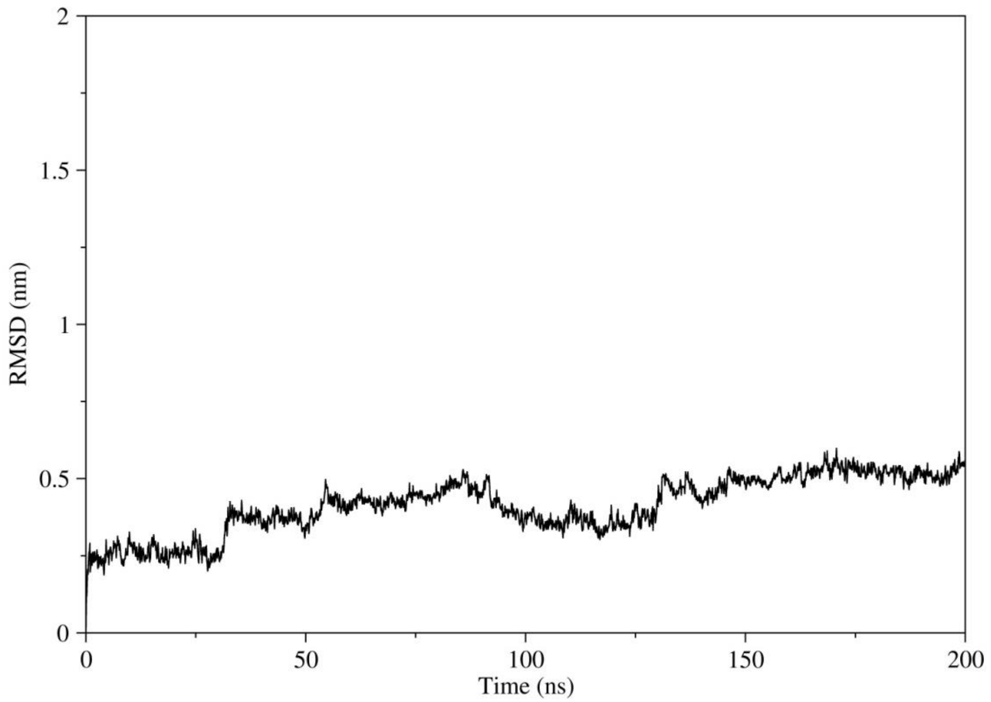

Fig. S5 Root mean square deviation (RMSD) of E200K-ligand **1** complex, calculated upon 200 ns of NPT trajectory

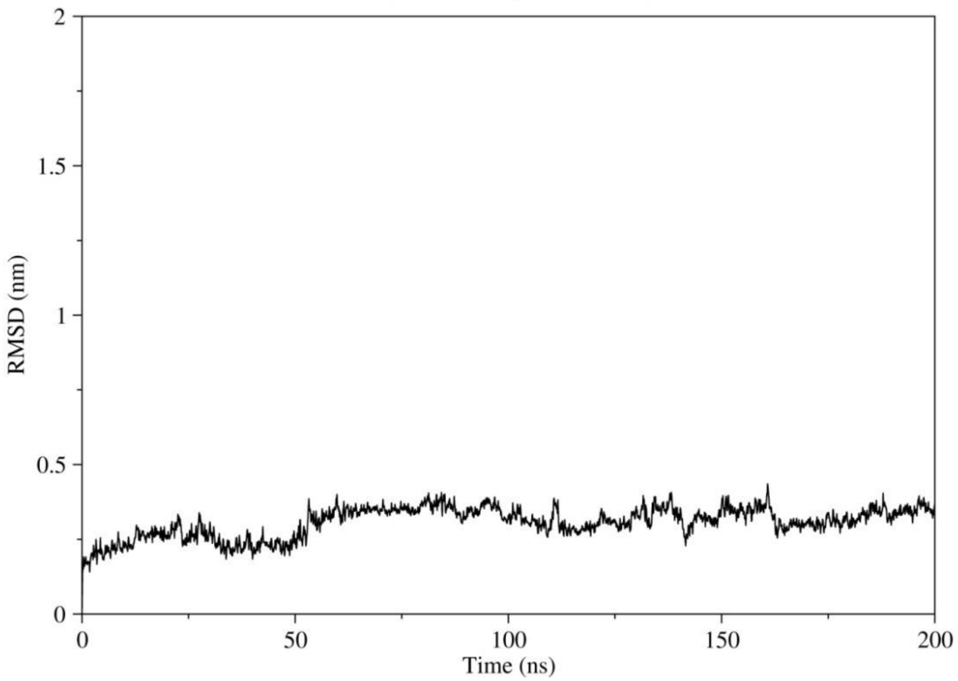

Fig. S6 Root mean square deviation (RMSD) of E200K-ligand **2** complex, calculated upon 200 ns of NPT trajectory

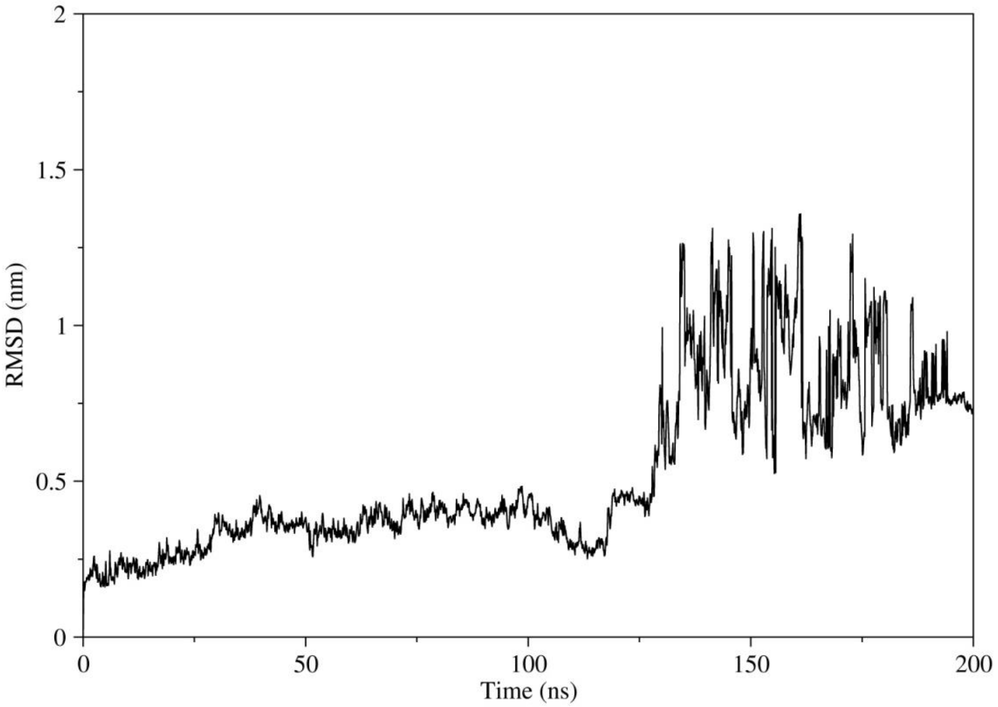

Fig. S7 Root mean square deviation (RMSD) of E200K-ligand **3** complex, calculated upon 200 ns of NPT trajectory

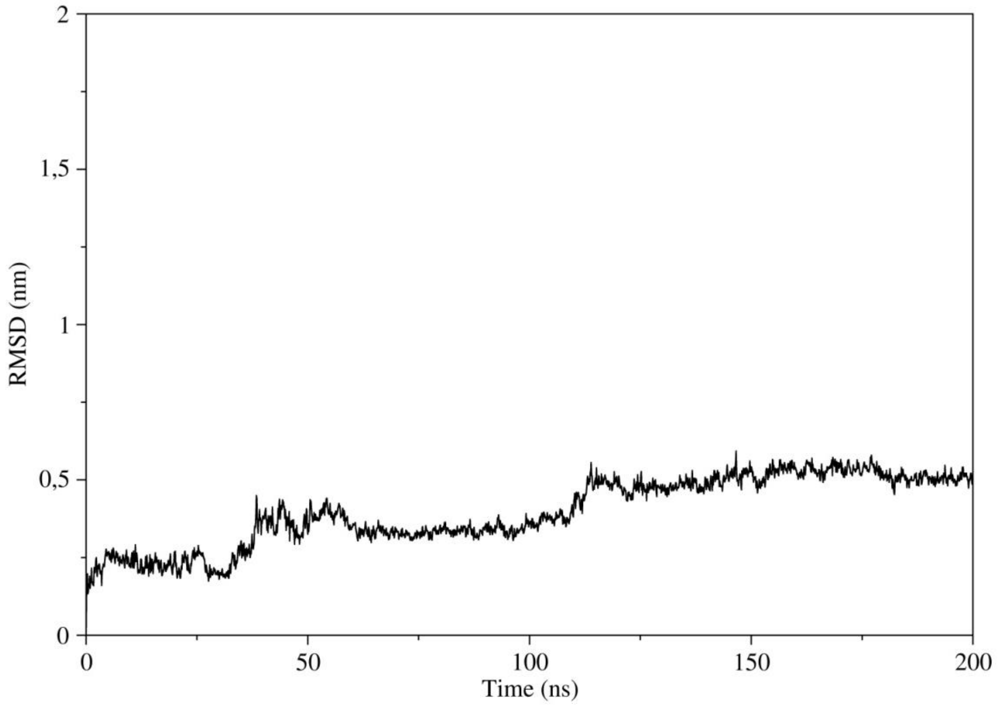

Fig. S8 Root mean square deviation (RMSD) of E200K-ligand **4** complex, calculated upon 200 ns of NPT trajectory

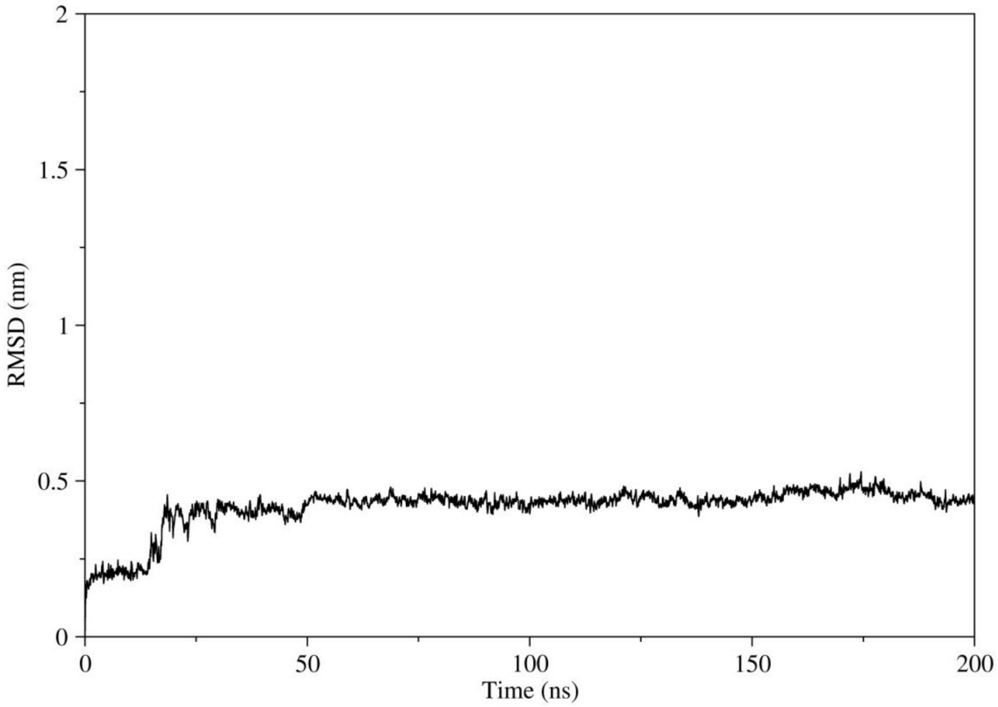

Fig. S9 Root mean square deviation (RMSD) of E200K-ligand **5** complex, calculated upon 200 ns of NPT trajectory

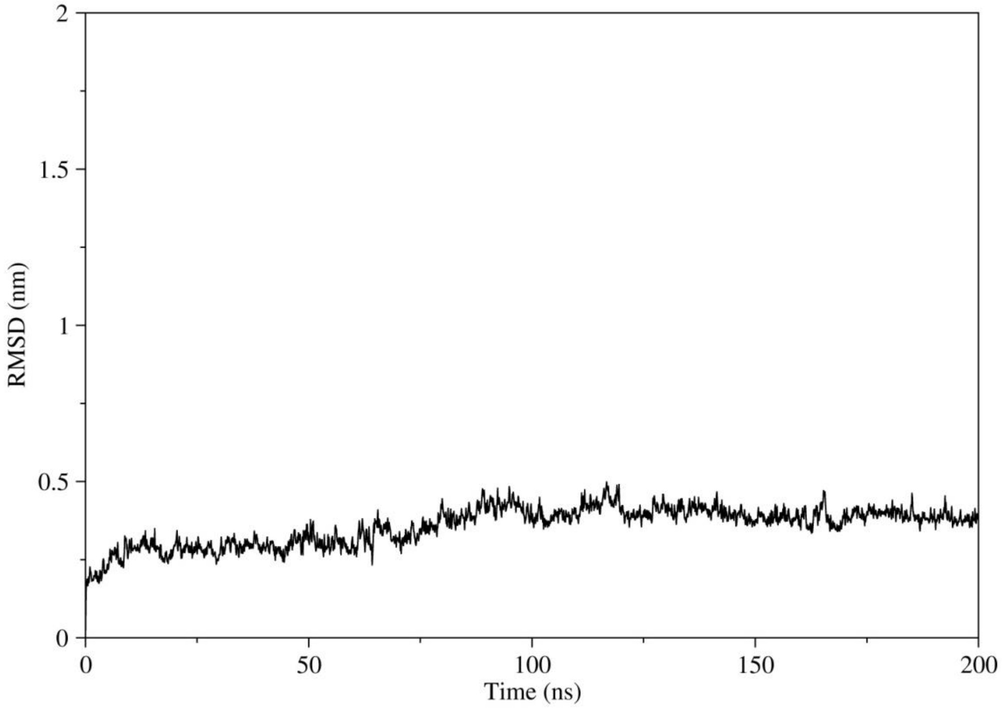

Fig. S10 Root mean square deviation (RMSD) of E200K-ligand **6** complex, calculated upon 200 ns of NPT trajectory

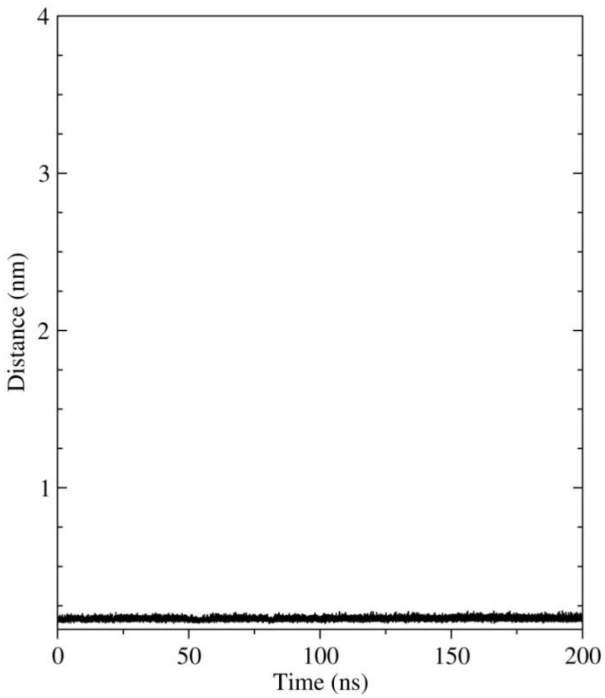

Fig. S11 Minimum distance between ligand **1** and E200K, computing along the 200 ns NPT trajectory

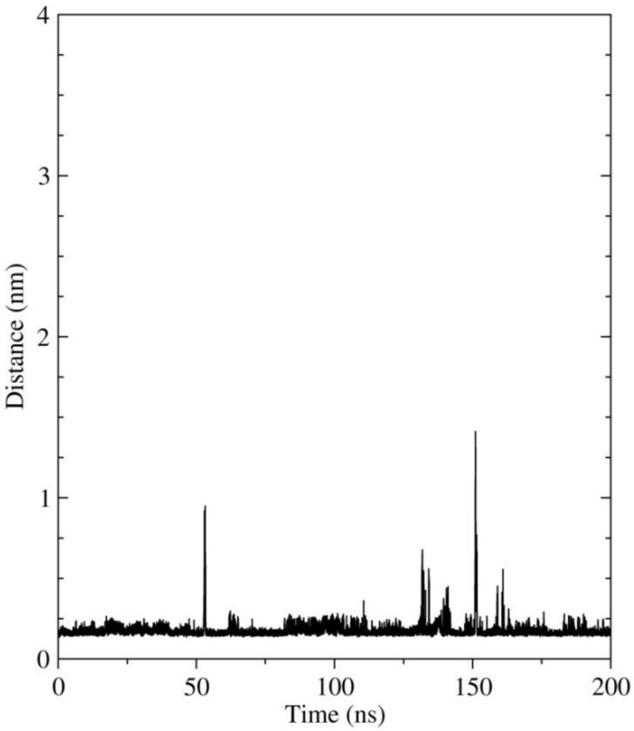

Fig. S12 Minimum distance between ligand **2** and E200K, computing along the 200 ns NPT trajectory

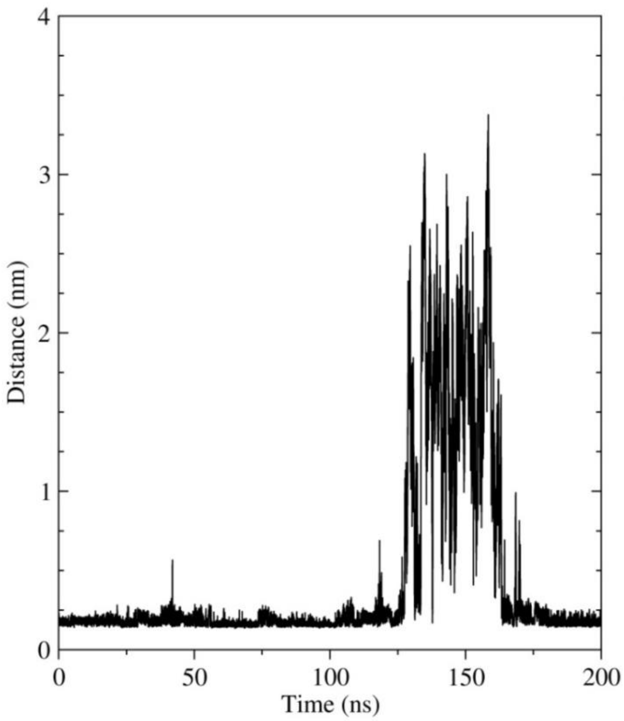

Fig. S13 Minimum distance between ligand **3** and E200K, computing along the 200 ns NPT trajectory

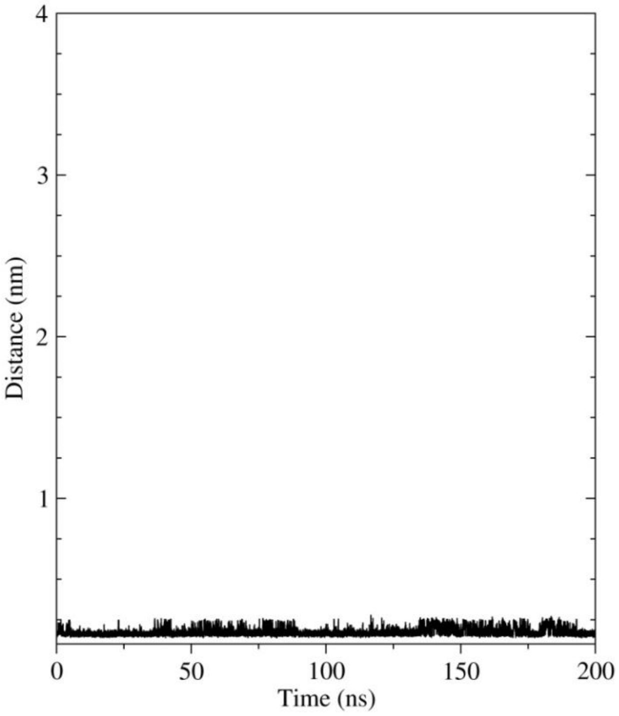

Fig. S14 Minimum distance between ligand **4** and E200K, computing along the 200 ns NPT trajectory

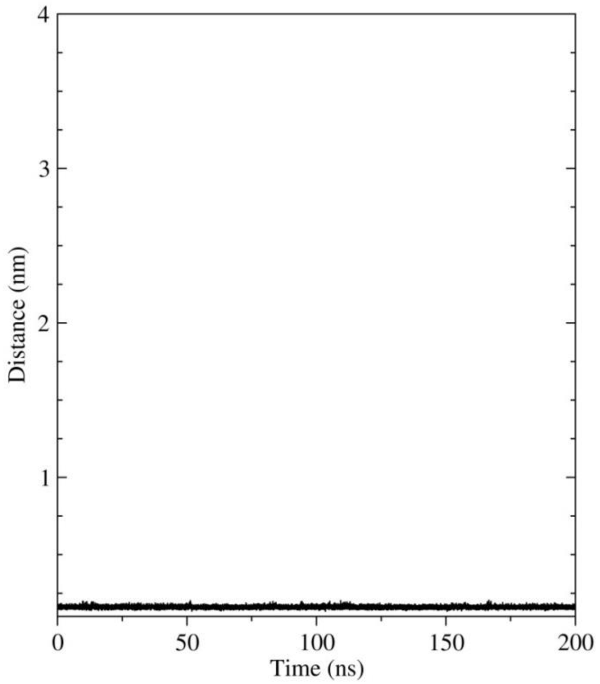

Fig. S15 Minimum distance between ligand **5** and E200K, computing along the 200 ns NPT trajectory

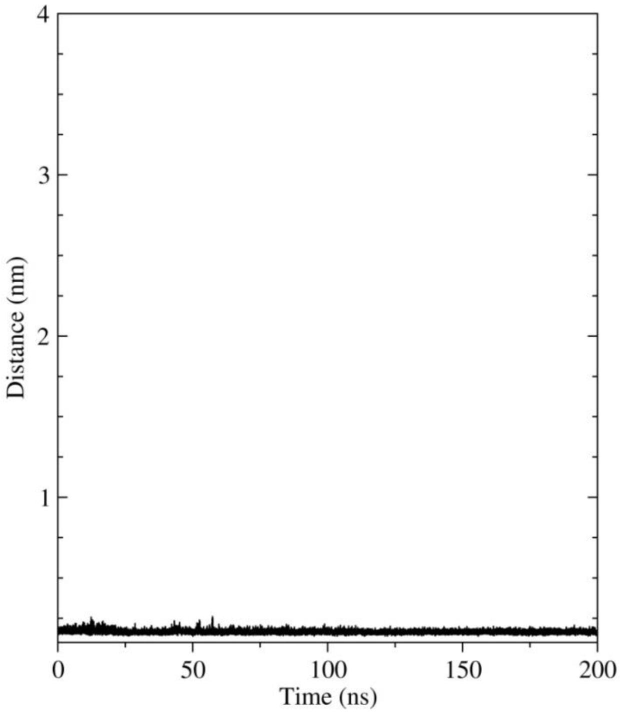

Fig. S16 Minimum distance between ligand **6** and E200K, computing along the 200 ns NPT trajectory

Table S4 Results of the clustering analyses of the MD trajectories of the complexes formed by E200K and the six selected virtual hits. A clustering cut-off of 0.19 nm was employed in the comparison of sampled MD snapshot based on the position of the ionizable residues

**Ligand**	**#Clusters**	**Population (%)**
1	4	87, 7, 3, 3
2	4	64, 29, 4, 3
3	3	78, 19, 3
4	4	50, 43, 4, 3
5	4	76, 17, 4, 3
6	3	82, 13, 5

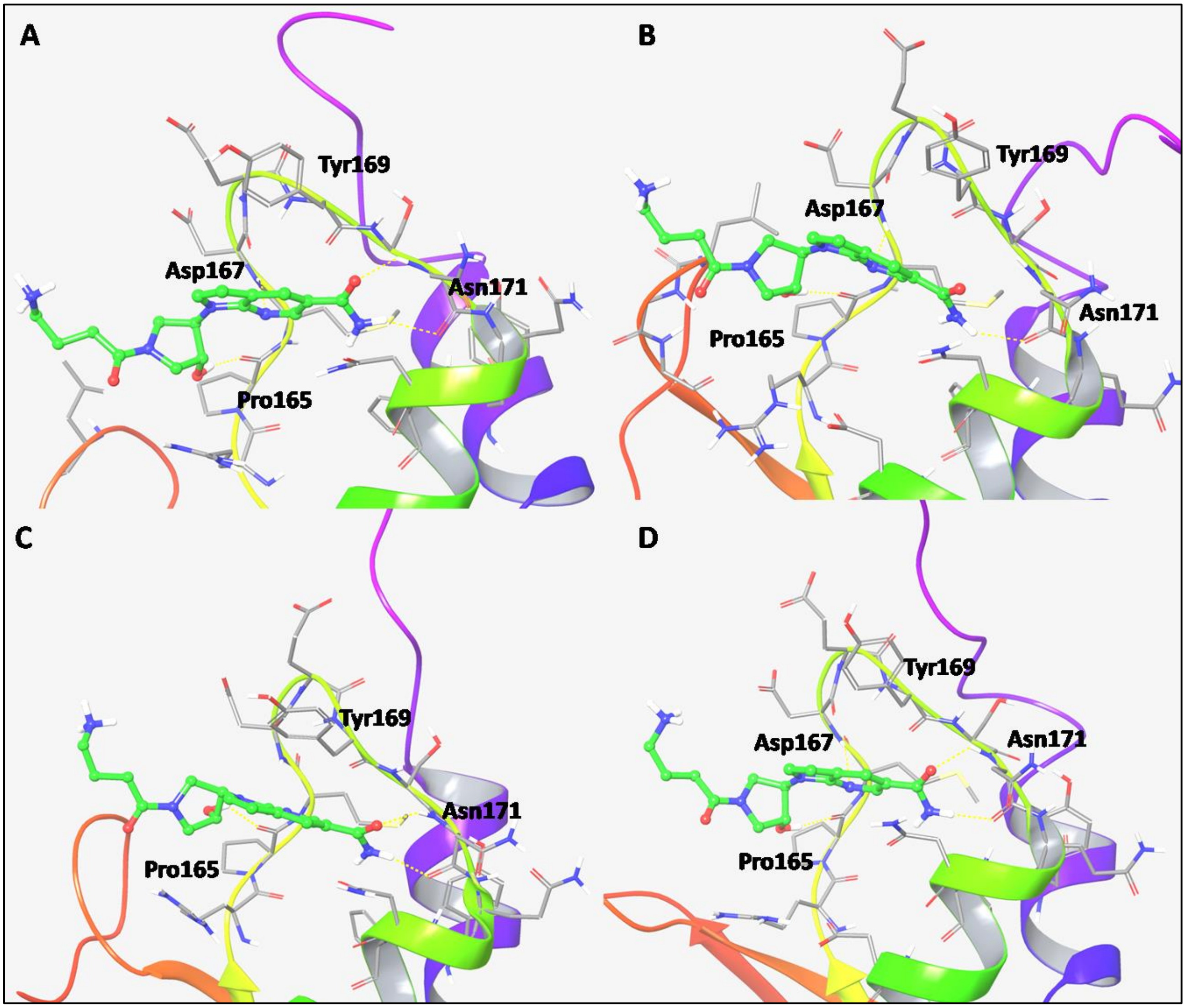

Fig. S17 Structures of the middle elements of the ligand **1** clusters: a) structure-1(87%), b) structure-2 (7%), c) structure-3 (3%) and d) structure-4 (3%). The hydrogen bonds are reported in dashed line (yellow)

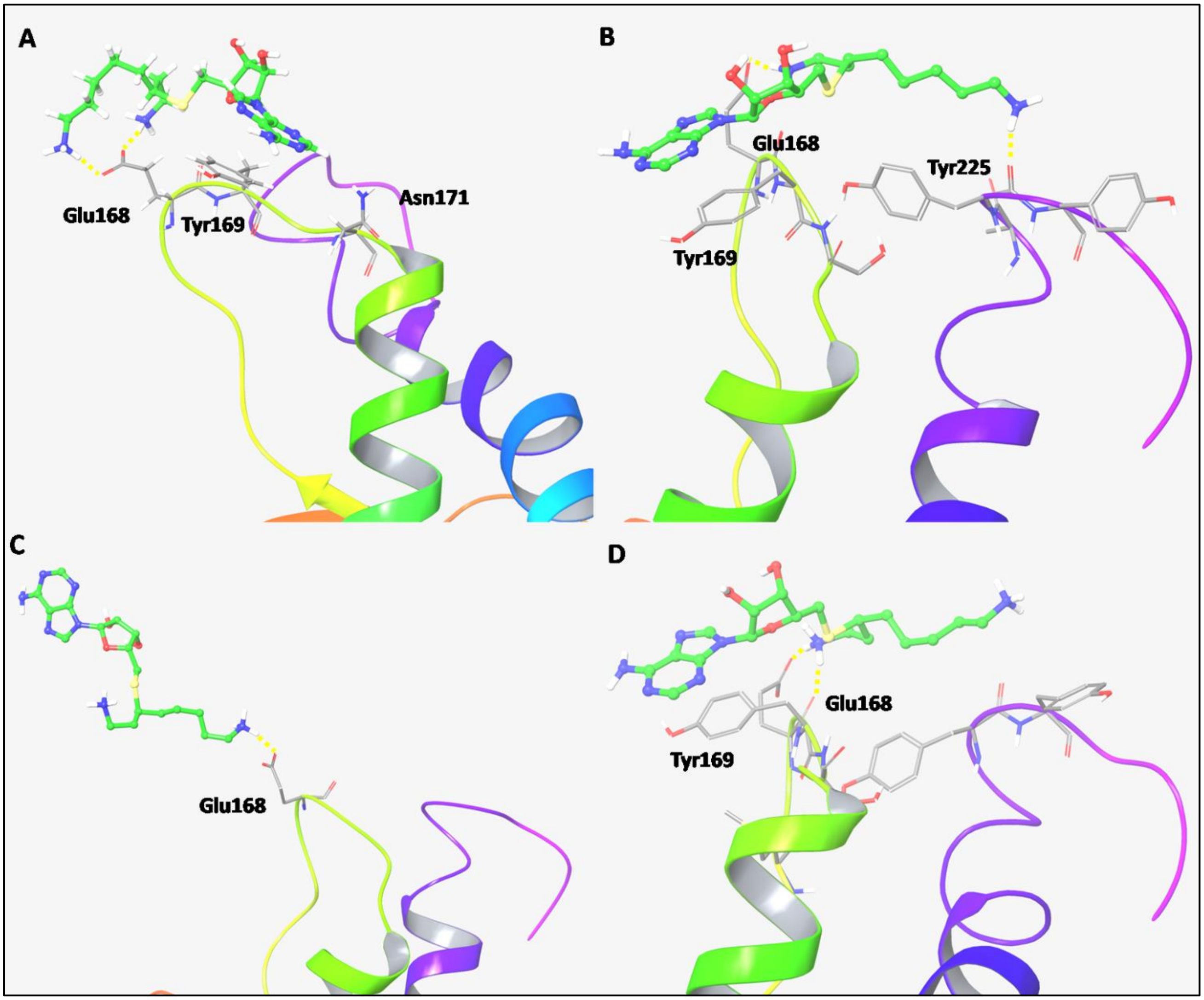

Fig. S18 Structures of the middle elements of the ligand **2** clusters: a) structure-1 (64%), b) structure-2 (29%), c) structure-3 (4%) and d) structure-4 (3%). The hydrogen bonds are reported in dashed line (yellow)

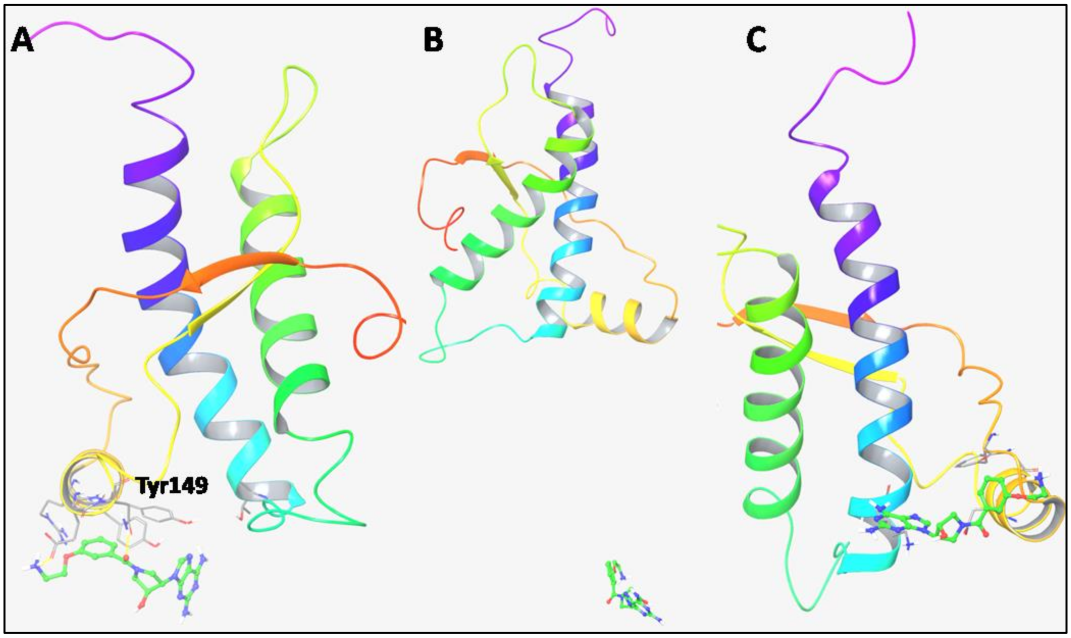

Fig. S19 Structures of the middle elements of the ligand **3** clusters: a) structure-1 (78%), b) structure-2 (19%) and c) structure-3 (3%). The hydrogen bonds are reported in dashed line (yellow)

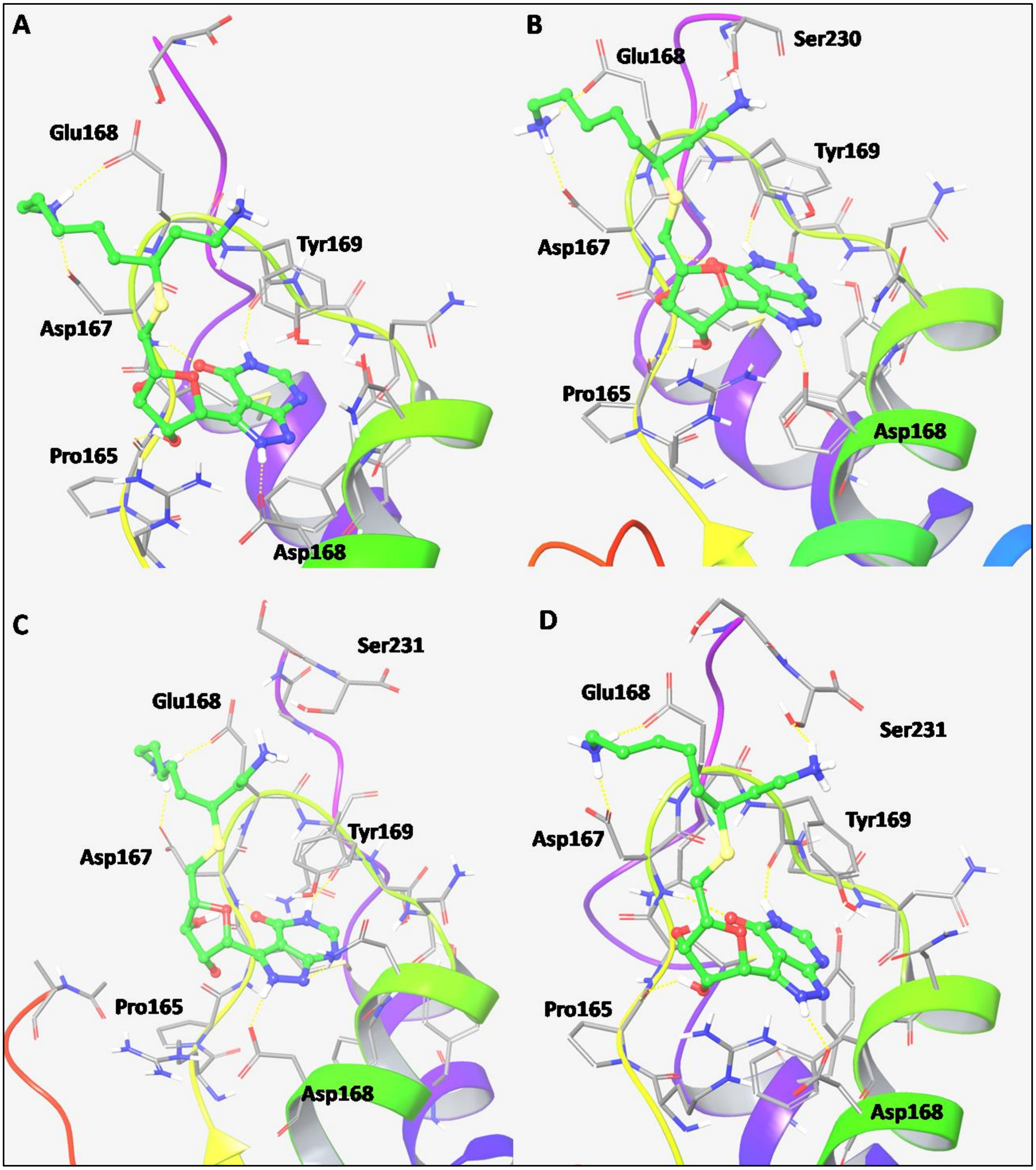

Fig. S20 Structures of the middle elements of the ligand **4** clusters: a) structure-1 (50%), b) structure-2 (43%), c) structure-3 (4%) and d) structure-4 (3%). The hydrogen bonds are reported in dashed line (yellow)

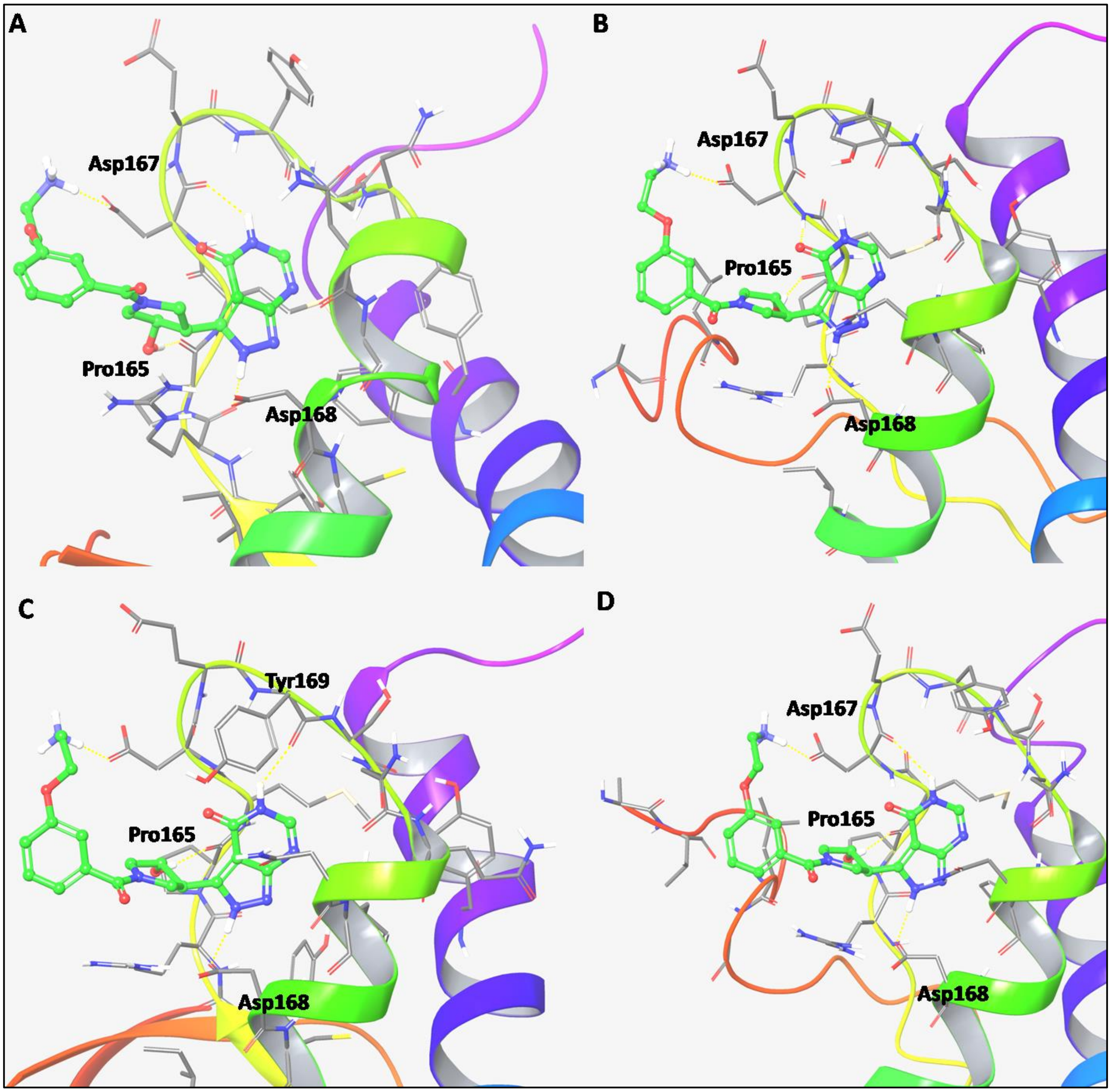

Fig. S21 Structures of the middle elements of the ligand **5** clusters: a) structure-1 (76%), b) structure-2 (17%), c) structure-3 (4%) and d) structure-4 (3%). The hydrogen bonds are reported in dashed line (yellow)

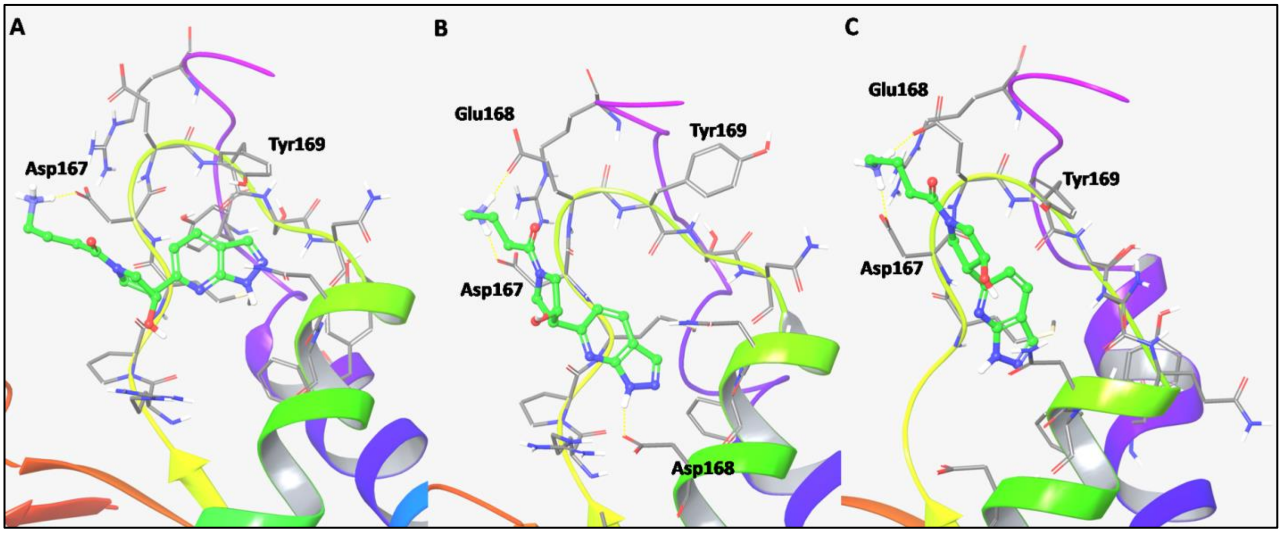

Fig. S22 Structures of the middle elements of the ligand **6** clusters: a) structure-1 (82%), b) structure-2 (13%) and c) structure-3 (5%). The hydrogen bonds are reported in dashed line (yellow)

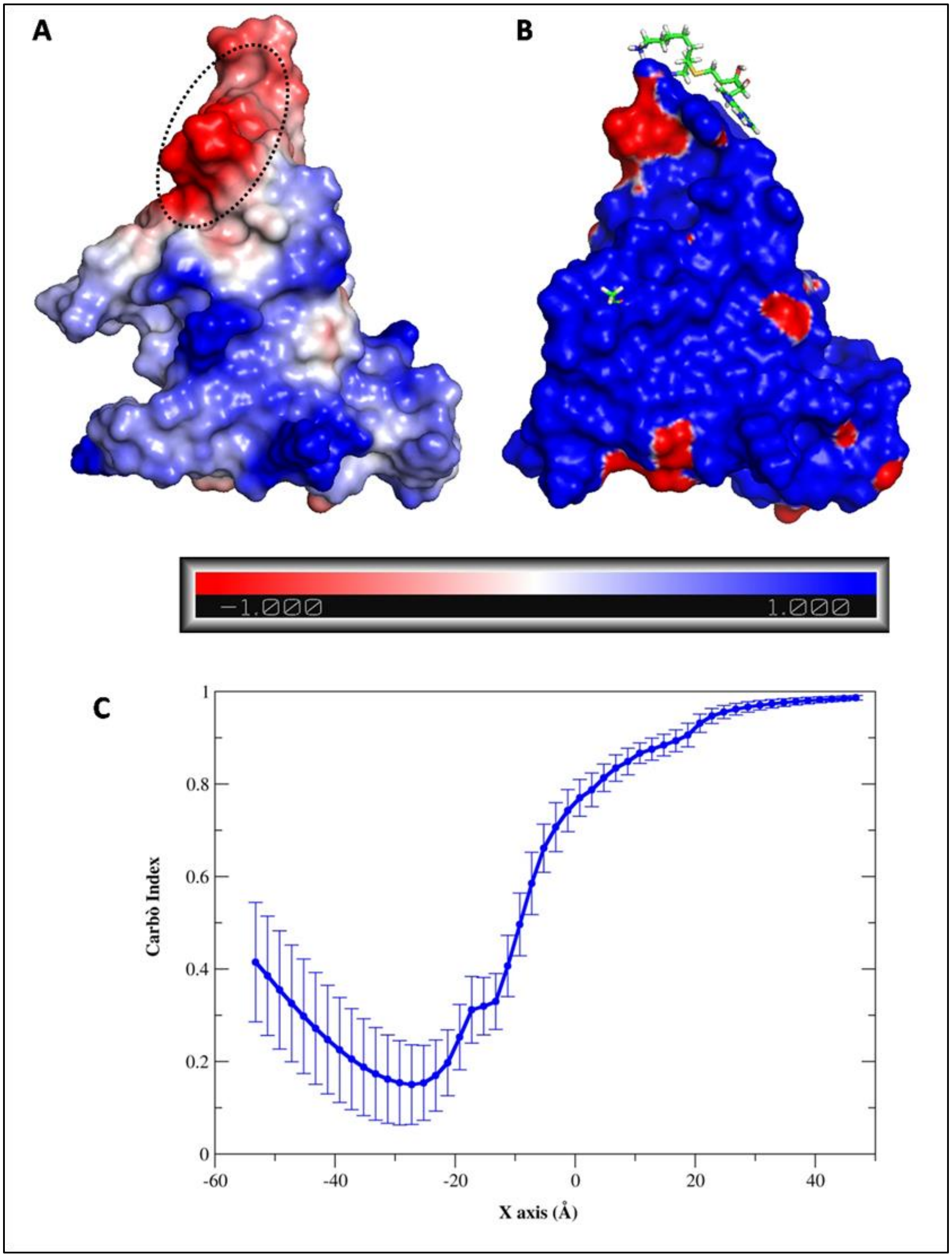

Fig. S23 The averaged MEP profiles of a) E200K-PrP and of b) E200K-ligand **2** complex. The negatively and positively charged regions are reported in red and blue, respectively. MEPs are computed and reported in arbitrary unit ranging in the -1.0 up to +1.0; c) Profiles of MEP similarity of E200K-ligand **2** complex.

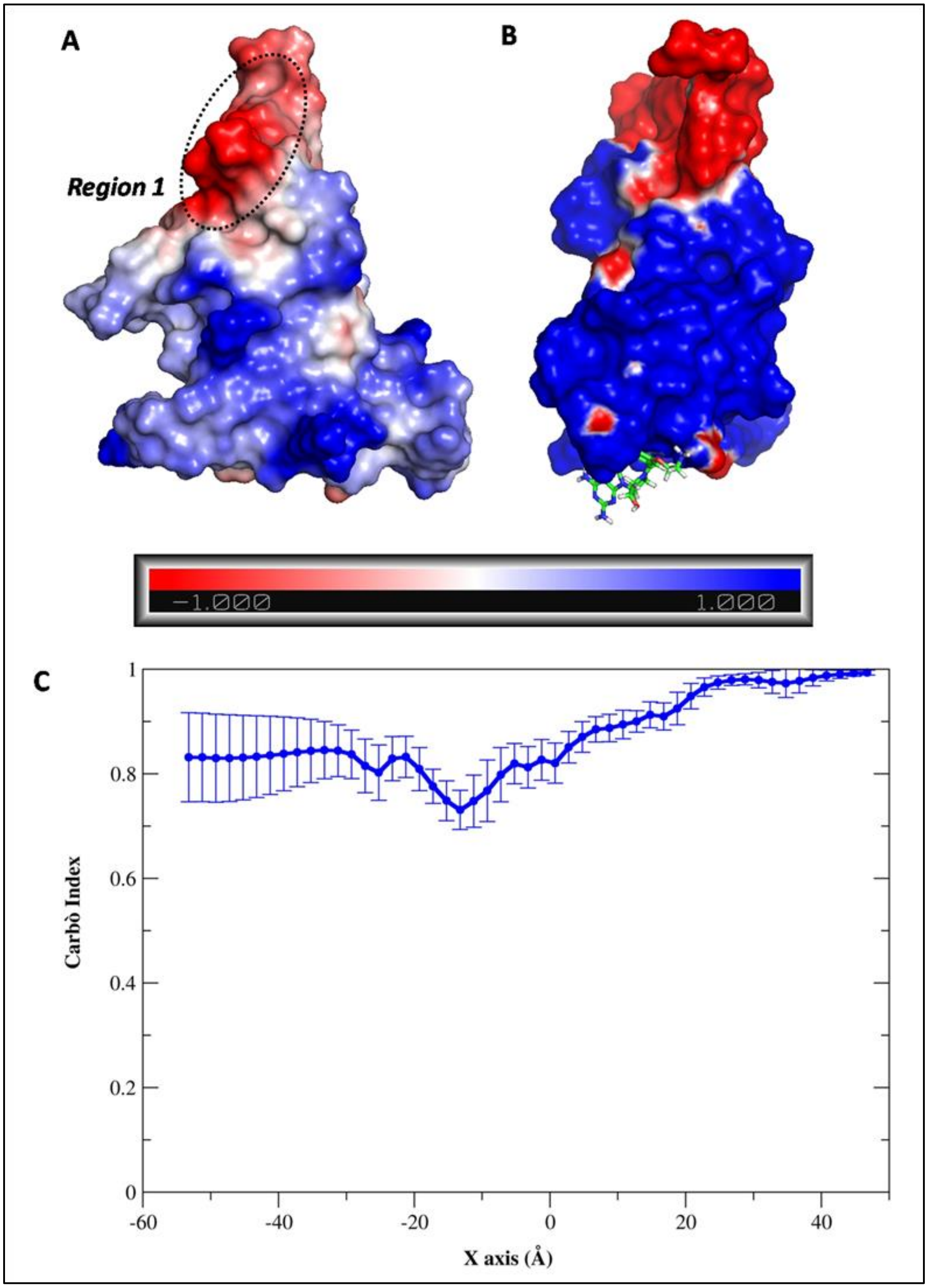

Fig. S24 The averaged MEP profiles of a) E200K-PrP and of b) E200K-ligand **3** complex. The negatively and positively charged regions are reported in red and blue, respectively. MEPs are computed and reported in arbitrary unit ranging in the -1.0 up to +1.0; c) Profiles of MEP similarity of E200K-ligand **3** complex

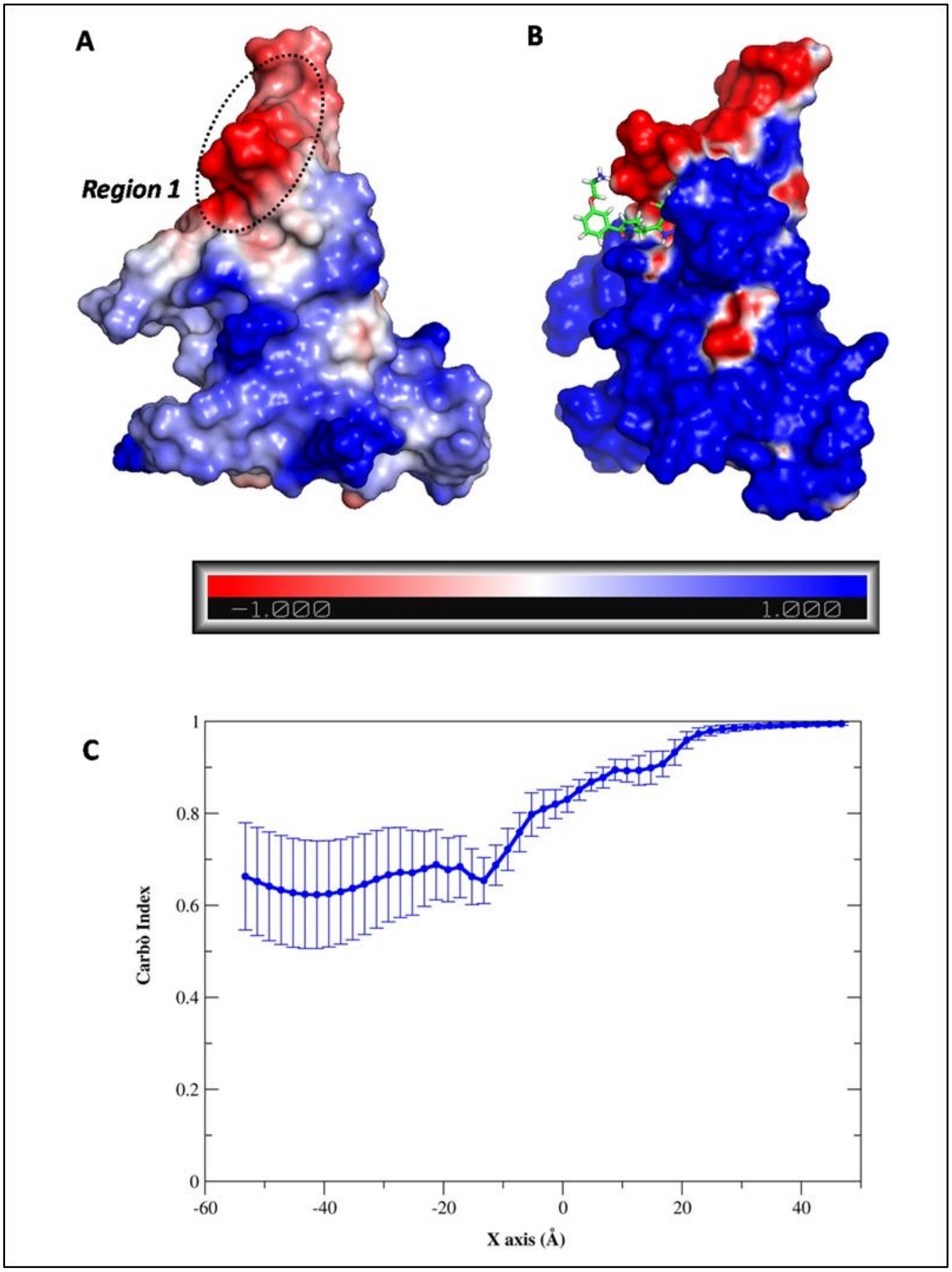

Fig. S25 The averaged MEP profiles of a) E200K-PrP and of b) E200K-ligand **5** complex. The negatively and positively charged regions are reported in red and blue, respectively. MEPs are computed and reported in arbitrary unit ranging in the -1.0 up to +1.0; c) Profiles of MEP similarity of E200K-ligand **5** complex

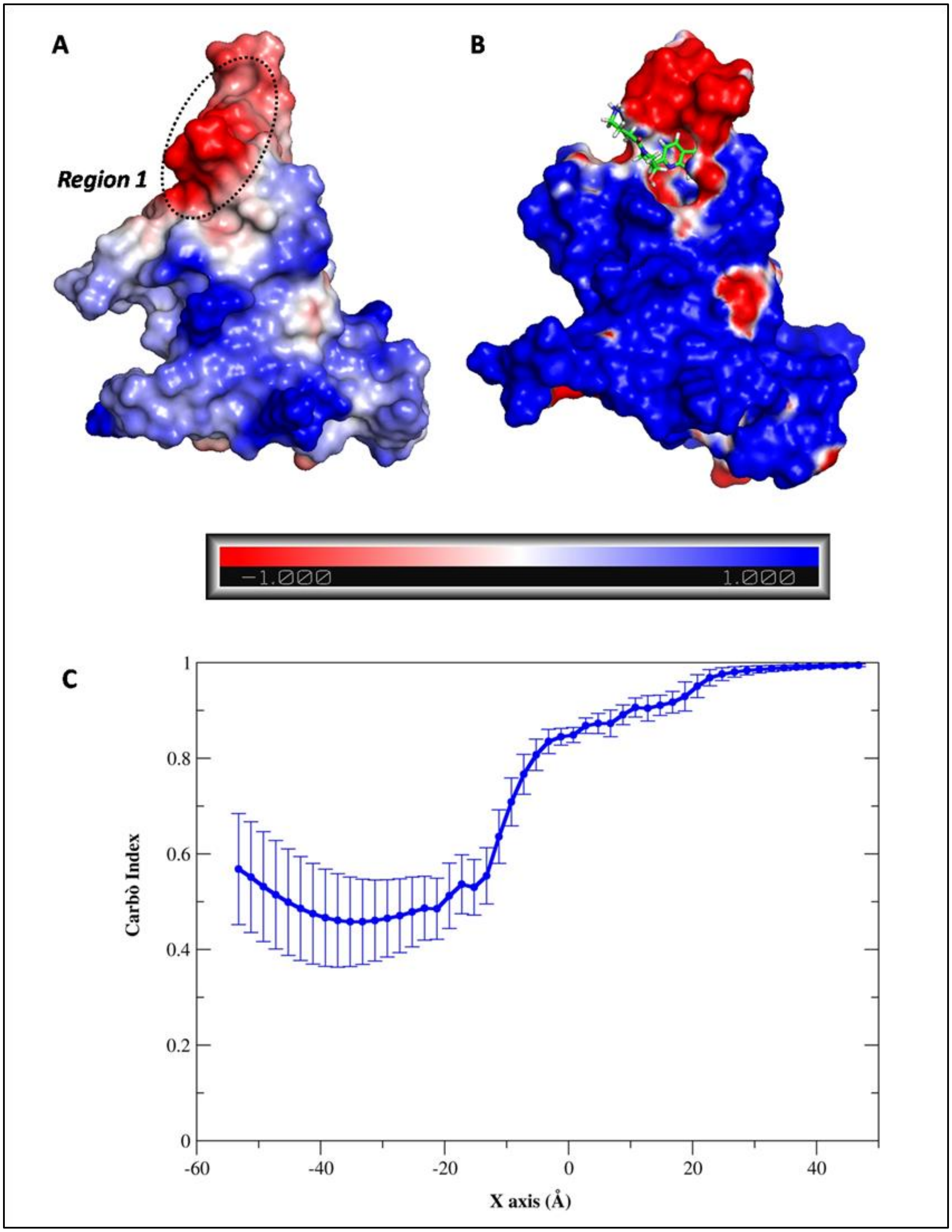

Fig. S26 The averaged MEP profiles of a) E200K-PrP and of b) E200K-ligand **6** complex. The negatively and positively charged regions are reported in red and blue, respectively. MEPs are computed and reported in arbitrary unit ranging in the -1.0 up to +1.0; c) Profiles of MEP similarity of E200K-ligand **6** complex

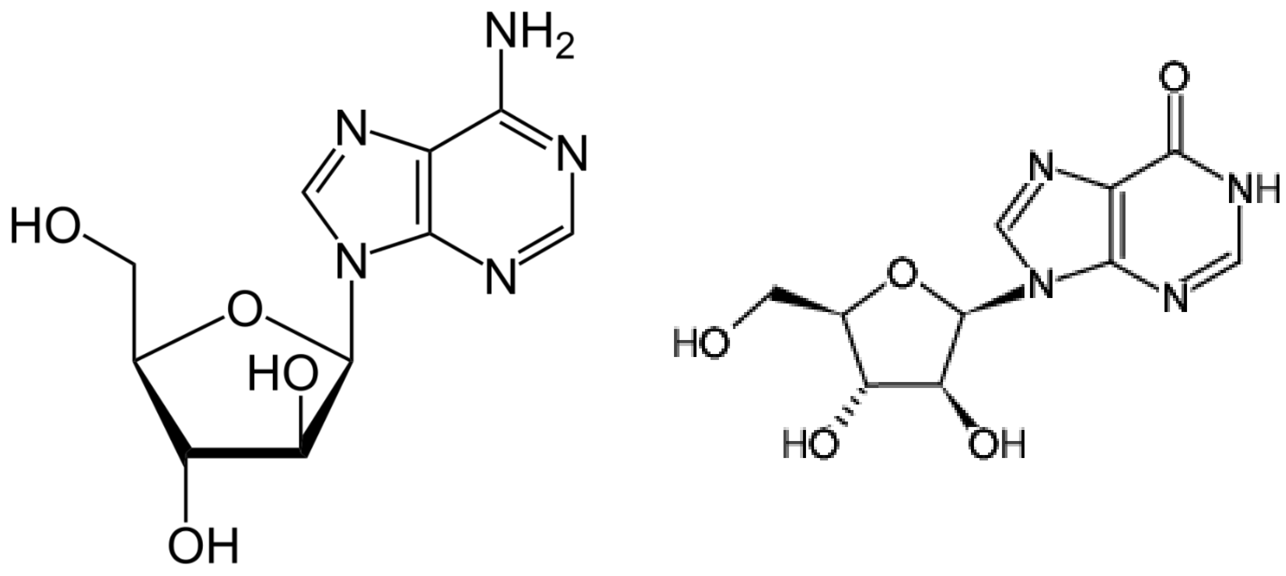

vidarabine **ara-HX**

Fig. S27 Chemical structures of vidarabine and its metabolite, ara-HX
